# Thermoresponsive Glycopolymers Based on Enzymatically Synthesized Oligo-β-Mannosyl Ethyl Methacrylates and *N*-Isopropylacrylamide

**DOI:** 10.1021/acs.biomac.0c01615

**Published:** 2021-05-07

**Authors:** Monica Arcos-Hernandez, Polina Naidjonoka, Samuel J. Butler, Tommy Nylander, Henrik Stålbrand, Patric Jannasch

**Affiliations:** † Centre for Analysis and Synthesis, Department of Chemistry, Lund University, S-221 00 Lund, Sweden; ‡ Physical Chemistry, Department of Chemistry, Lund University, S-221 00 Lund, Sweden; § Department of Biochemistry and Structural Biology, Department of Chemistry, Lund University, S-221 00 Lund, Sweden

## Abstract

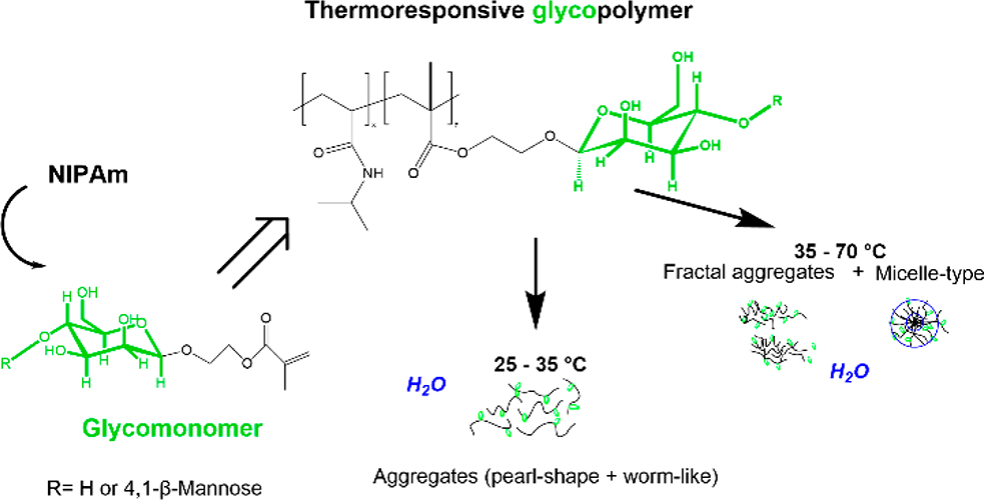

We present here a series of thermoresponsive glycopolymers in the form of poly(*N*-isopropylacrylamide)*-co-*(2-[β-manno[oligo]syloxy] ethyl methacrylate)s. These copolymers were prepared from oligo-β-mannosyl ethyl methacrylates that were synthesized through enzymatic catalysis, and were subsequently investigated with respect to their aggregation and phase behavior in aqueous solution using a combination of ^1^H NMR spectroscopy, dynamic light scattering, cryogenic transmission electron microscopy (TEM), and small-angle X-ray scattering (SAXS). The thermoresponsive glycopolymers were prepared by conventional free radical copolymerization of different mixtures of 2-(β-manno[oligo]syloxy)ethyl methacrylates (with either one or two saccharide units) and *N*-isopropylacrylamide (NIPAm). The results showed that below the lower critical solution temperature (LCST) of poly(NIPAm), the glycopolymers readily aggregate into nanoscale structures, partly due to the presence of the saccharide moieties. Above the LCST of poly(NIPAm), the glycopolymers rearrange into a heterogeneous mixture of fractal and disc/globular aggregates. Cryo-TEM and SAXS data demonstrated that the presence of the pendant β-mannosyl moieties in the glycopolymers induces a gradual conformational change over a wide temperature range. Even though the onset of this transition is not different from the LCST of poly(NIPAm), the gradual conformational change offers a variation of the temperature-dependent properties in comparison to poly(NIPAm), which displays a sharp coil-to-globule transition. Importantly, the compacted form of the glycopolymers shows a larger colloidal stability compared to the unmodified poly(NIPAm). In addition, the thermoresponsiveness can be conveniently tuned by varying the sugar unit-length and the oligo-β-mannosyl ethyl methacrylate content.

## 1 Introduction

Glycopolymers are synthetic biobased polymers that have sugar groups as pendant moieties. They attract great attention because of their function as biomimetic analogues of glycolipids and glycoproteins.^1,2^ Glycopolymers through their sugar moieties can potentially bind specifically to proteins, which are responsible for several interactions at the cellular level such as cell recognition and cell adhesion.^3,4^ As such, glycopolymers can be used as biomaterials for drug delivery, tissue engineering, and biosensors and in medicine.^5^ Responsive polymers are polymers that undergo conformational changes when exposed to an external stimuli (temperature, pH, light, etc.). This type of polymer is valuable in applications where such changes are advantageous under certain conditions,^6^ for example, in food, cosmetics,^7^ paints, and oil recovery,^8^ as well as in biomedical applications for injectable hydrogels and controlled drug release.^9,10^ In particular, temperature responsive polymers undergo a lower critical solution temperature (LCST) transition, resulting in a conformational coil-to-globule transformation upon exceeding a certain temperature.^11^ At this temperature, the polymer chain contracts as water becomes a poor solvent for the polymer.^12^ Hence, the polymer changes its character from hydrophilic to more hydrophobic, and is therefore prone to aggregation. One of the most widely studied thermoresponsive polymers is poly(*N*-isopropylacrylamide) [poly(NIPAm)] due to its temperature dependent phase transition in aqueous solution at ∼32 °C, close to body temperature. The LCST behavior of poly(NIPAm) is frequently modulated by copolymerization with hydrophilic or hydrophobic monomers such as 2-hydroxy ethyl methacrylate (HEMA).^13^

Glycopolymers that can undergo conformational changes under certain conditions, such as changes in temperature, are of great interest. Sugar moieties have previously been incorporated into thermoresponsive glycopolymers.^14−16^ For example, thermoresponsive double hydrophilic diblock glycopolymers (DHDG) from poly(NIPAm) and an α-linked mannose-containing acrylate and galactose-functionalized have been reported.^15^ Recently, a set of temperature-switchable glycopolymers from NIPAm and α-mannose ligands were synthesized.^17^ These remarkable glycopolymers are mostly based on glycomonomers that contain monosubstituted pendant sugar units and have commonly been synthesized through multistep pathways.

We have previously shown that β-mannanases, a type of glycoside hydrolase, can catalyze the synthesis of 2-(β-manno[oligo]syloxy) ethyl methacrylates (M_*n*_EMAs) (i.e., mannosyl-EMA [M_1_EMA] and mannobiosyl-EMA [M_2_EMA]) that were subsequently used to synthesize glycopolymers.^18^ The enzyme-catalyzed synthesis of glycomonomers presents several advantages compared to traditional chemical synthesis, including the avoidance of cumbersome protection–deprotection steps and toxic chemicals, as well as the possibility to use low temperature.^18^ Although other glycopolymers containing enzymatically synthesized monomers (glycomonomers) have been previously prepared,^2,4,19^ our glycomonomers (M_*n*_EMAs) feature an equatorial (β) linkage at the anomeric position with the acceptor and between mannose residues, making these monomers quite unique. This linkage occurs widely within plants, hemicelluloses, and storage glycans but has been shown to be very difficult to synthesize chemically.^20^ We were only able to find one report on such a linkage featured in a glycopolymer, which was demonstrated to be a potent inhibitor resistant to exo-α-mannosidase digestion with enhanced affinity for concanavalin A.^21^ Hence, we believe that our glycopolymers can find applications in similar fields.

In the present work, the transglycosylation capacity of the β-mannanase *TrMan5A* was utilized for glycomonomer synthesis in a one-pot reaction in water at 37 °C using locust bean galactomannan as the donor glycan.^18^ This provided a mixture of functionalized acrylate monomers bearing one to three β-mannose units. Subsequently, we were able to separate and purify the different M_*n*_EMAs for polymer synthesis at microscale. While the enzymatic reaction shows good yields and is seamlessly scalable, the separation and purification steps require further optimization to increase the yields and sample amounts available after isolation. Here, we used the available amounts of monomers in combination with NIPAm to synthesize thermoresponsive glycopolymers by conventional free radical polymerizations (FRP) in water at ambient temperature. FRP is the most common type of polymerization in the industry, accounting for around 40–45% of all industrial polymers^22^ due to its simplicity and tolerance to impurities which reduces costs. Several studies have demonstrated that glycopolymers synthesized via FRP possess adequate affinity to biological targets, although they are polydisperse.^1,23^ A brief summary of potential applications of glycopolymers synthesized with FRP has been reported by Babiuch and Stenzel.^24^ We expect that in the future, the use of FRP will increase the viability of these applications at large scale. We anticipate that the introduction of a biobased molecule with mannose moieties would lead to a certain degree of affinity for lectins, viruses, and/or toxins, and that such interactions can be tuned for specific applications. Here, we provide the basis for such applications by providing viable synthesis routes, molecular characteristics, thermoresponse, and solution structure and behavior.

In order to influence the solubility properties and the transition temperature of the copolymers in aqueous solution, we varied the molar fraction and the sequence of the sugar units. As mentioned above, M_*n*_EMAs are glycomonomers with a hydrophilic biodegradable mannosylated part and a polymerizable acrylate part. By incorporating the glycomonomers in copolymers with NIPAm, we aimed to modify the thermoresponsive behavior of NIPAm thanks to the mannosyl moieties. We then studied the thermoresponsive behavior of the glycopolymers using dynamic light scattering (DLS), combined with ^1^H nuclear magnetic resonance (NMR) spectroscopy, small-angle X-ray scattering (SAXS), and cryogenic transmission electron microscopy (cryo-TEM).

## 2 Experimental Section

### 2.1 Materials

Sodium acetate (molecular biology grade), acetic acid, diethyl ether (anhydrous, ≥99.7%, with 1 ppm BHT as inhibitor), hydroquinone (HQ, ≥99%), acetonitrile (ACN, ≥99.9%, HPLC gradient grade), 2,5-dihydrobenzoic acid (DHB), *N*-isopropylacrylamide (NIPAm), deuterated water (D_2_O), 2,2′-azobis(isobutyronitrile) (AIBN), potassium persulfate (KPS), and *N,N,N*′*,N*′-tetramethylethylenediamine (TEMED), HPLC grade ethanol, 2,2′-azobis(isobutyronitrile) (AIBN), diethyl ether, 2-hydroxyethyl methacrylate (HEMA) 97% containing 200 ppm hydroquinone (HQ) were all obtained from Sigma-Aldrich (St. Louis, MO, USA). Low-viscosity locust bean gum (LBG, >94% (dry weight basis)) was supplied by Megazyme (Bray, Ireland) (LOT 150901a) (galactose:mannose ratio, 24:76). All chemicals were used as received except for HEMA, which was passed through an alumina column prior to polymerization to remove the inhibitor.^18^

#### TrMan5A

Glycoside hydrolase family 5 β-mannanase Man5A from *Trichoderma reesei* (TrMan5A) was prepared as previously described.^25^ An amount of 20 mg of freeze-dried enzyme was dissolved in 50 mM sodium acetate buffer (50 mM), pH 5.3. The solution was concentrated thrice, from 10 to 0.2 mL, in Sartorius VivaSpin 20 columns with a 10 kDa cutoff (Sartorius, Göttingen, Germany), refilling the column with fresh buffer between each concentration step. The concentration was performed at 4 °C, 5000 rpm, 30 min, in VivaSpin 20 centrifugal concentrators (10000 MWCO PES membrane).

### 2.2 Methods

#### Synthesis of 2-(β-Manno[oligos]yloxy) ethyl methacrylates) [M_*n*_EMAs]

Enzymatic synthesis of the target glycomonomers, M_*n*_EMAs, denoted with *n* = 1 and 2, respectively, was done using the method developed in our previous work, except the volume of the reaction was 500 mL instead of 50 mL.^18^ In short, the reaction was carried out in 500 mL 30 mM sodium acetate buffer, pH 5.3. The donor substrate was galactomannan in the form of low-viscosity locust bean gum (LBG) (3 w.v%) and the acceptor substrate, HEMA (20 vol %). The reaction was catalyzed by 0.2 μM of the β-mannanase TrMan5A at 37 °C, for 48 h, in a stirred, covered glass beaker, with intermittent sampling to enable reaction progression analysis. A volume of 250 mL of the enzymatic synthesis mixture was used for purification and isolation of the individual glycomonomers, M_*n*_EMAs, as previously described.^18^^1^H and ^13^C shift assignment has been extensively described previously.^18^ We observed differences in the upscaled reactions (50 mL vs 500 mL). In particular, the recovery yield of the glycomonomers after purification and isolation was lower in the latter. Consequently, the amount available of the purified glycomomoners M_1_EMA and M_2_EMA was of 18.7 mg and 29.5 mg, respectively (as determined by ^1^H NMR). Therefore, due to the small amounts of the purified monomers available, we designed polymerizations at microscale in an NMR tube. We later discovered that we incurred losses of the glycomonomers during the different purification and isolation steps prior to the polymerizations. For example, a proportion of the glycomonomers coprecipitated together with saccharides when processing the sample after the enzymatic reaction. Optimization of recovery and purification methods on larger scale are subject of ongoing work. Nevertheless, we were able to prepare several samples on the microscale. Detailed descriptions on the preparation of the monomers prior to polymerization are described in Supporting Information (SI) part A.

#### Synthesis of Poly(*N*-isopropylacrylamide)*-co-*(2-[β-manno[oligo]syloxy] ethyl methacrylate)s [poly(NIPAm*-co-*M_*n*_EMA)s]

A total of seven different polymers were prepared in solution via conventional free radical polymerization as previously described:^18^ two glycopolymers based on M_1_EMA, three based on M_2_EMA, and two reference samples. We will refer to the glycopolymers using the template PNM*X*-*YY* where *X* = 1 or 2 for M_1_EMA and M_2_EMA, respectively, and *YY* = % molar content of the glycomonomer after polymerization (*Y*_polymer_ in Tables 1 and S1). The two reference samples were a homopolymer of NIPAm (PN) and a copolymer of NIPAm and HEMA (PNEMA-22, where EMA= HEMA). Details of the experimental design of the polymerizations are found as SI part A and Table S1.

**Table 1:** Calculated Yields, Polymer Compositions, and Thermoresponsive Behavior of the Glycopolymers [poly(NIPAm*-co-*M_*n*_EMA)s].^a^

polymer type	sample designation	*Y*_feed_^b^ M_*n*_EMA (or HEMA) [mol/mol]	*Y*_polymer_^c^ M_*n*_EMA (or HEMA) [mol/mol]	NIPAm conversion^d^ [mol %]	M_*n*_EMA (or HEMA) conversion^e^ [mol %]	*T*_onset_^f^ [°C]	LCST_NMR_^g^ [°C]
Poly(NIPAm*-co-*M_1_EMA)	PNM1–08	0.07	0.08	95	∼100	34.1 ± 0.1	40.1 (0.01)
	PNM1–18	0.18	0.18	92	∼100	34.1 ± 0.1	39 (0.02)
Poly(NIPAm*-co-*M_2_EMA)	PNM2–03	0.02	0.03	83	∼100	34.5 ± 2.8	45.1 (0.01)
	PNM2–16	0.15	0.16	96	∼100	35.4 ± 0.3	40.1 (0.004)
	PNM2–18	0.18	0.18	97	∼100	34.9 ± 0.1	42.1 (0.02)
Poly(NIPAm)^h^	PN	-	-	∼100	-	34.7 ± 0.01	34.7 (0.01)
Poly(NIPAm*-co-*EMA)	PNEMA-22	0.14	0.22	75	94	30.2 ± 0.3	32.3 (0.03)
	PNEMA-22P					31.4 ± 1.4	36.9 (0.02)

a All yields and compositions were calculated using ^1^H NMR data (see SI, part J for details).

b Molar fraction of the monomer (M_*n*_EMA or HEMA) measured in the reaction solution before polymerization with ^1^H NMR data (±2 mol %).

c Molar fraction of the monomer (M_*n*_EMA or HEMA) incorporated in the polymer determined in the polymerization solutions with ^1^H NMR immediately after stopping the reaction (±2 mol %).

d Conversion of the monomer of NIPAm determined with ^1^H NMR.

e Conversion of the monomer (M_*n*_EMA or HEMA) determined with ^1^H NMR.

f Temperature at which the thermal transition starts as defined by parameter *x*_o_ in Equation S1.

g As noted by ^1^H NMR, it was defined as the temperature at which the intensity of the chemical shift studied is 50% of the intensity measured at 25 °C (i.e., *I*_LCSTNMR_ = 0.5*I_25_, *I* = NMR intensity), and was obtained by resolving Equation S1 for *y* = 0.5. Within parentheses we report the standard error of estimate (SSE) of the fitted model. A detailed explanation of how the parameters were derived can be found in SI part C.

h *M*_n_ = 636 800 g/mol; *M*_w_= 785 500 g/mol.

**Scheme 1:**
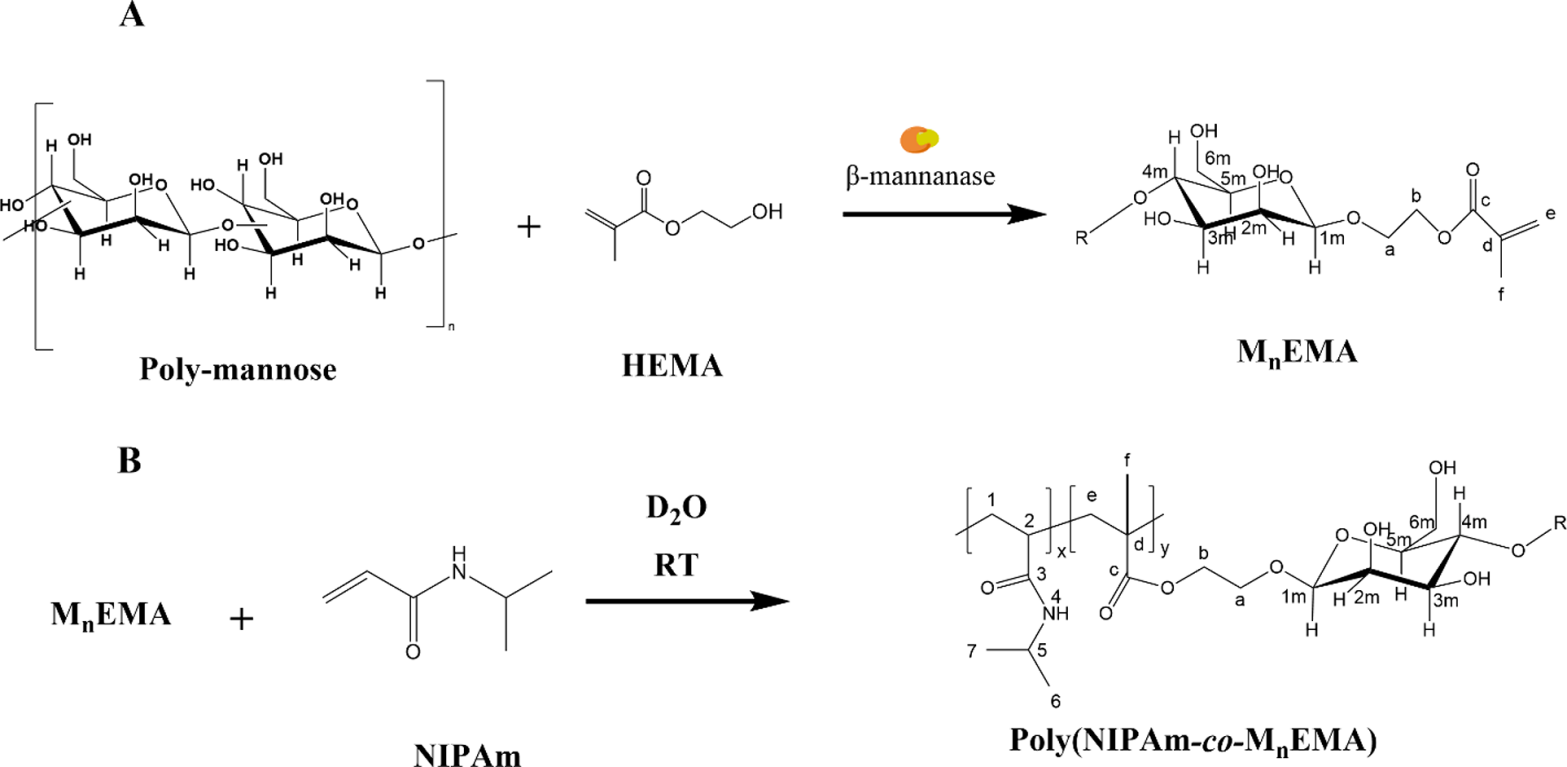
Synthesis of Poly(NIPAm*-co-*M_*n*_EMA)s (A) Enzymatic synthesis of oligo-β-mannosyl ethyl methacrylates (M_*n*_EMAs). (B) Radical copolymerization of NIPAm and M_*n*_EMAs in D_2_O. M_1_EMA: R = H and M_2_EMA R = β-4,1-mannose.

The copolymers of NIPAm and M_*n*_EMAs and the homopolymer of NIPAm were synthesized in D_2_O with KPS as initiator and TEMED as accelerator. The reactions were performed at room temperature in an NMR spectroscopy tube as previously reported.^18^ Since the amounts available were limited, we decided to use an NMR tube in order to directly characterize the glycopolymer solutions by NMR spectroscopy, thus avoiding too many unnecessary sample transfer steps that could result in sample loss. Sample PNEMA-22 was prepared in ethanol with AIBN as initiator at 60 °C similar to the literature.^26^Scheme 1 summarizes the synthesis of the glycomonomers and their subsequent copolymerization with NIPAm.

Two samples (PNM1–18 and PNM2–16) were nominally prepared with the same mass ratio of monomers (NIPAm:M_*n*_EMA) in order to compare the effect of the length of the sugar moieties given the same weight content in the synthesized copolymer. Samples PNM1–08 and PNM1–18, on one hand, and samples PNM2–03 and PNM2–18, on the other hand, were prepared with the same glycomonomer (M_1_EMA or M_2_EMA, respectively) in different molar concentrations in order to investigate the effect of the comonomer ratio on the thermoresponsive behavior. Finally, PNEMA-22 was a reference sample synthesized and studied to investigate the effect of the acrylate part on the LCST of poly[NIPAm] in relation to the effect induced by the sugar moieties.

#### NMR Spectroscopy

^1^H and ^13^C spectra of the glycomonomers before polymerization and of solutions of the poly(NIPAm-*co*-M_*n*_EMA) samples were recorded on a Bruker Avance III spectrometer (Bruker, Billerica, MA, USA) at 500.17 MHz for ^1^H and at 125.77 MHz for ^13^C. Quantification of monomers was performed as previously described.^18^ A second method for quantification was used to confirm the results from the first. This second method was done using the ERETIC2 quantification tool available in the NMR processing software Topspin (Bruker Biospin, 2018) as previously described.^27^ The method can be used with an external standard. In our studies, we used a stock of purified HEMA as external standard. Five solutions with the concentrations 5, 11, 21, 32, and 54 mg/L were prepared from this stock. A full description of the NMR data acquisition can be found in SI part B. The error in our calibration method for determining the concentration was estimated to be ±2 mol %.

#### Study of the LCST Transitions of Poly(NIPAm-*co*-M_*n*_EMA)s by ^1^H NMR Spectroscopy

^1^H NMR spectra were recorded as described in SI part B at a range of temperatures, first heating from 25 °C to 50–65 °C, and then cooling from 50 to 65 to 25 °C. In general, all spectra of a single sample were taken sequentially in the same run. For each sample, the samples were gradually heated to the selected temperature and equilibrated during 6 min if the change in temperature was smaller than 3 °C, and during 10–15 min if the change was above 3 °C.

To study the LCST transitions from the acquired spectra, the intensities of selected chemical shifts at different temperatures were scaled to the intensity at 25 °C and plotted against temperature. A five-parameter sigmoidal model was then fitted to the data from which two parameters were derived, i.e., the onset of the LCST, *T*_onset_, defined as the temperature at which the thermal transition starts (i.e., temperature at the maximum slope), and a parameter that we have named LCST_NMR_, which was obtained by resolving the sigmoidal equation (Equation S1) for when the intensity was half of the initial intensity at 25 °C (*I* = 0.5). This definition of LCST has been used in studies of similar thermoresponsive polymers.^16^ Details of the model fitting can be found in SI part C.

#### Polymerization Kinetics

^1^H NMR spectra were recorded during the polymerization of samples PNM1–18 and PNM2–18 to gain insight into the reaction kinetics. Consequently, ^1^H spectra were acquired at different time intervals. This was possible because the polymerizations were conducted at room temperature and inside an NMR tube. Hence, the possible effect of interference due to sample convection at temperatures higher than room temperature was negligible. The reaction was followed for the first 9 h after initiation.

#### Dynamic Light Scattering (DLS)

The size of the particles as a function of temperature was followed with a Zetasizer Nano ZS (Malvern Instruments Ltd., Worshestershire, UK) at a set angle of 173° using the noninvasive backscatter technology. The instrument was equipped with a 4 mW He–Ne laser with a 632.8 nm wavelength and an Avalanche photodiode detection (APD) unit. The obtained correlation functions were analyzed using the cumulants method available in the Malvern software. The samples were diluted 10 times with D_2_O (∼1 mg/mL) and filtered through 0.45 μm pore-size hydrophilic filter to remove dust and larger particles. The correlation functions were recorded at temperatures from 25 to 70 °C in 2 °C steps. The samples were equilibrated for 3 min at every temperature prior to the measurement.

In addition, dynamic and static light scattering (DLS and SLS) measurements were performed with sample PNM2–16 on an ALV/DLS/SLS-5022F, CGH-8F-based compact goniometer system (ALV-GmbH, Langen, Germany) with a 22 mW He–Ne laser (632.8 nm) light source. The instrument was equipped with an automatic attenuator, controlled via software. A cuvette with the sample was placed in a cell housing filled with a refractive index matched liquid (*cis*-decahydronaphtalene). DLS measurements were performed at temperatures 25–45 °C with 2 °C step at 90° angle, while SLS was measured at 25 and 45 °C and at the detector angles of 30–140°. From these measurements, we derived hydrodynamic radius (*R*_h_) and a radius of gyration (*R*_g_) at 25 and 45 °C and calculated a shape parameter ρ = *R*_g_/*R*_h_. The treatment of the data from DLS and SLS is described in detail in SI part D.

#### Small Angle X-ray Scattering (SAXS)

SAXS measurements were performed using the Ganesha SAXS Lab instrument (SAXS Lab, Denmark). The instrument was equipped with a GeniX 3D 30 W Cu X-ray tube (Xenocs) and a 2D 300 K Pilatus detector (Dectris). The measurements were acquired with a pinhole collimated beam with the detector positioned asymmetrically to yield *q-*range of 0.012–0.67 Å^–1^ and 0.003–0.21 Å^–1^. The sample-to-detector distance was 480 and 1540 mm, respectively.

The magnitude of the scattering vector is defined by *q* = (4 π sin θ)/λ, where the wavelength λ equals 1.54 Å (Cu Kα wavelength) and θ is half of the scattering angle. The temperature in the analysis chamber was controlled using a Julabo T Controller CF41 from Julabo Labortechnik GmbH (Germany). Samples were measured at the initial concentration (∼10 mg/mL) and two temperatures, 25 and 50 °C, with the equilibration time of 30 min before each measurement. The obtained scattering curves were corrected for background scattering, and data from different detector distances were combined to cover the desired *q-*range. The reduced data was evaluated with SasView^28^ and fitted to the Unified Exponential/Power-law model developed by Beaucage that describes fractal-like behavior of polymers in solution and a polymer micelle model for some cases as described in the Results.^29−31^ The scattering intensity of fractal objects can be described with the fractal model, *I*(*q*) ∼ *q*^*–d*^ where the exponent *d* can be obtained by fitting this model to the scattering curve.^28^ In order to fit such data, a corrected Beaucage model is often applied.^29−31^ This model describes fractal polymer chains that consist of flexible cylinders and gives two radii of gyration (*R*_g_).^31^ The largest *R*_g_ is determined from the low-*q* or Guiner regime and represents the overall size of the particle, whereas the second radius of gyration is obtained from the *q* region where the curve shows transition in the slope and describes the size of a subunit of the polymer chain (*R*_sub_). The latter radius can be converted into persistence length (*L*_p_), which is an indication of the chain stiffness and can be calculated as^32,33^
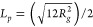
1

#### Cryogenic Transmission Electron Microscopy (cryo-TEM)

The polymerization solutions had high viscosity and were therefore diluted 10-fold before imaging with cryo-TEM (∼1 mg/mL). For sample imaging, a 4 μL drop of the sample was placed on a lacey carbon-coated Formvar grid (Ted Pella Inc., Redding, CA, USA) and gently blotted with a filter paper to create a thin film. The grid was then prepared for imaging using an automatic plunge-freezer system (Leica Em GP, Leica Microsystems, Wetzlar, Germany) with the environmental chamber operated at 25 and 50 °C. The specimen was then vitrified by rapid plunging of the grid into liquid ethane (−183 °C). Thereafter, samples were stored in liquid nitrogen (−196 °C) and transferred into the microscope using a cryo transfer tomography holder (Fischione, Model 2550, E.A. Fischione Instruments, Inc., Corporate Circle Export, PA, USA). The grids were examined with a Jeol JEM-2200FS transmission electron microscope (JEOL, Tokyo, Japan) equipped with a field-emission electron source, a cryo-pole piece in the objective lens, and an in-column energy filter (omega filter). Zero-loss images were recorded under low-dose conditions at an acceleration voltage of 200 kV on a bottom-mounted TemCam-F416 camera (TVIPS-Tietz Video and Image Processing Systems GmbH, Gauting, Germany) using SerialEM.

#### Size Exclusion Chromatography (SEC)

The polymers were analyzed by size exclusion chromatography (SEC) to determine the weight-average molecular weight (*M*_w_) and number-average molecular weight (*M*_n_) in three different setups with different mobile phases. In the first system, a sample volume of 20 μL (1 mg/mL) was injected on a TSKgel G4000PW_XL_ column (TOSOH Bioscience GmbH, Griescheim, Germany) connected to a chromatography system (Waters 600E System Controller, Waters, Milford, MA, USA), using RI (Waters 2414 Differential Refractometer) and UV detection (Waters 486 Tunable Absorbance Detector) at 234 nm. Deionized water was used as eluent at a flow rate of 0.5 mL/min. The column was calibrated with dextran standards of 50, 150, 270, and 410 kDa (Fluka Chemie AG, Buchs, Swizerland). In the second system, the samples were injected into a Waters Alliance HPLC with UV and RI detectors with two columns connected in series, GE Healthcare Superdex 30 Increase 10/300 + Superdex 200 Increase 10/300 at room temperature in 0.1 M NaOH as mobile phase (0.5 mL/min). Finally, in the third system the samples were first freeze-dried for 1 week prior to the SEC analysis. Samples were injected into an Agilent 1100/1200 Infinity HPLC System with GPC column PSS GRAM calibrated with polystyrene standards. Three PSS GRAM GPC columns of dimensions 300 × 8.00 and particles size of 10 μm were used. The columns had different porosities 3000 (S/N 90610005), 1000 (S/N 9111012), and 30 Å (S/N 9031611). The mobile phase used was *N,N*-dimethylacetamide (DMAc) with 5 g/L lithium bromide (LiBr). The system was calibrated with poly(methyl methacrylate) standards in the range 0.266–1820 kDa.

## 3 Results and Discussion

### 3.1 Synthesis of Thermoresponsive Glycopolymers [poly(NIPAm-*co-*M_*n*_EMA)s]

**Figure 1:**
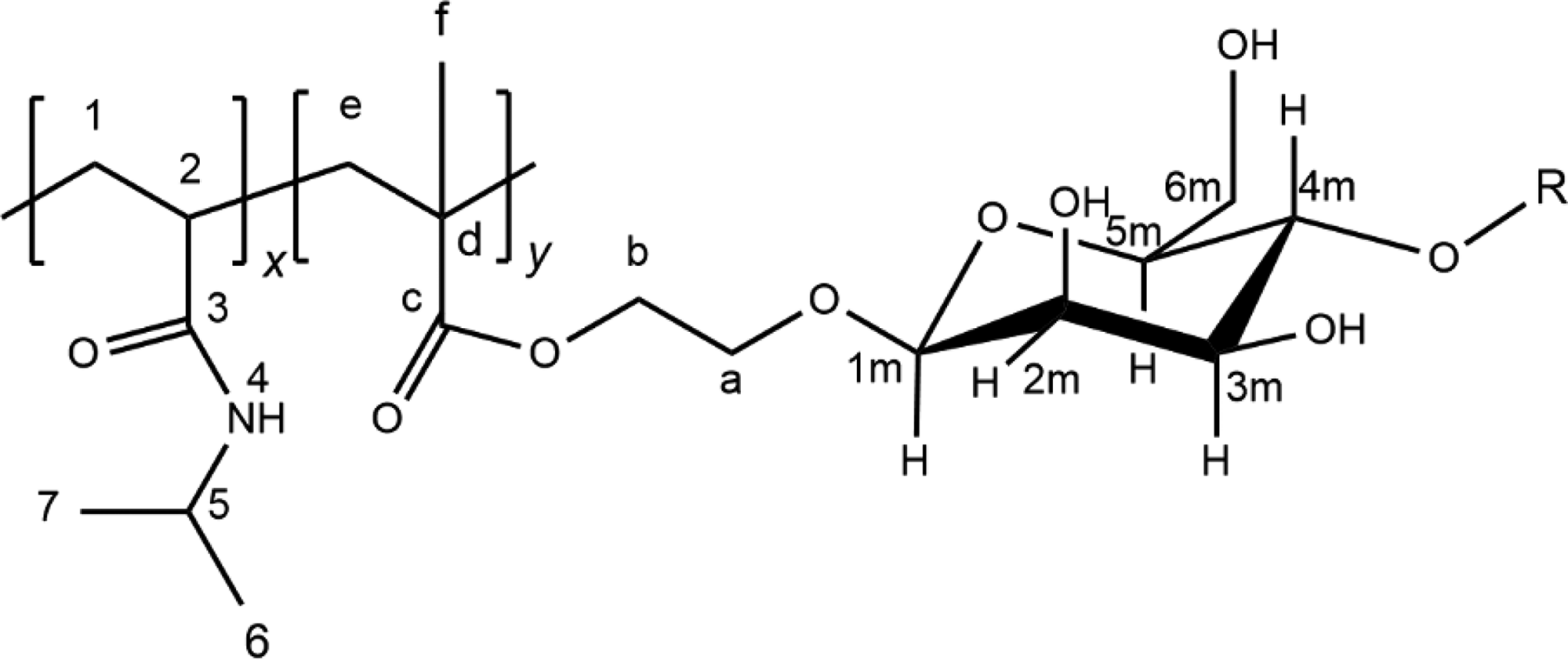
Expected molecular structure of the glycopolymers poly(NIPAm-*co*-M_*n*_EMA)s, indicating the structure of a β-mannosyl unit. M_1_EMA: R = H and M_2_EMA: R = β-4,1-mannose.

The enzymatically synthesized M_*n*_EMAs were used in conventional free radical polymerizations (FRP) with NIPAm to yield copolymers with the general structure shown in Figure 1, as confirmed by ^1^H and ^13^C NMR spectra. A representative example is given in Figures S1 and S2 in the SI for sample PNM2–18. We have previously fully resolved the ^1^H NMR spectra of homopolymers based on M_1_EMA and M_2_EMA^18^ and the spectrum of poly(NIPAm) is well-known.^34,35^

The polymerizations were monitored by measuring monomer conversions over time using ^1^H NMR data. For all the poly(NIPAm*-co-*M_*n*_EMA)s, the NMR data acquired during the reaction showed that the conversions given in Table 1 were reached after 2 h of reaction and there was no further increase after this time. The synthesis results, based on data extracted from ^1^H NMR spectroscopy measurements, are summarized in Table 1. The detailed analysis of the ^1^H NMR data over time is presented in SI part J.

The polymerizations showed conversions of NIPAm above 92 mol %, as determined from the ^1^H NMR spectra. The conversion of the glycomonomer was estimated to be ∼100 mol %. Already in our previous work, we demonstrated that these glycomonomers are readily polymerizable and able to reach full conversions in FRP.^18^ Furthermore, the ^13^C spectra showed no signal at 127.01 ppm that would correspond to the acrylate double bond in M_*n*_EMA (Scheme 1C-e).^18^ Only signals unequivocally assigned to unconverted NIPAm monomer at 130.14 and 126.72 ppm were found in that region. In addition, the signal at 17.4 ppm that originated from the −CH_3_ of M_*n*_EMA disappeared upon polymerization, which we have previously reported (see Figure S2).^18^ Sample PNM2–03 had a lower molar monomer concentration compared to the rest of the samples. Nevertheless, relatively high conversions were also achieved in this polymerization, even without the addition of the accelerator. Sample PNEMA-22 reached a conversion of 75 mol % for NIPAm. It has previously been shown that HEMA polymerizes faster than NIPAm in similar systems.^36^ Analysis of the copolymers by size exclusion chromatography (SEC) was attempted, initially using water as the mobile phase. The chromatograms showed very weak and broad signals, and all the samples eluted at the exclusion volume (Figure S6). This indicated the presence of aggregates in the solutions. We have previously described the same outcome for homopolymers synthesized from the same glycomonomers.^18^ Next, we attempted SEC analysis using 0.1 NaOH M as the mobile phase, but results similar to the water system were obtained. A third attempt was made using a system with DMAc/LiBr as the mobile phase employing freeze-dried samples as previously described. Regrettably, the freeze-dried samples were not fully soluble in the mobile phase (DMAc/LiBr), except for the ply[NIPAm] homopolymer (PN) which was estimated to have *M*_n_ = 636 800 g/mol and *M*_w_ = 785 500 g/mol (Table 1). Hence, we were not able to determine the molecular weights of the glycopolymers. However, we note that Furyk and co-workers have shown that the polydispersity and *M*_w_ had little effect on the LCST of finely mass fractionated samples of poly(NIPAm) as long as the polymer *M*_w_ was above 50 kDa, and only slight deviations were noted with lower-molecular-weight samples.^37^

### 3.2 Thermoresponsive Transitions of poly(NIPAm-*co-*M_*n*_EMA)s by NMR Spectroscopy and DLS

^1^H NMR spectroscopy of thermoresponsive polymers in D_2_O solutions provides important insights into the thermoresponsive behavior on the molecular level.^6,38,39^ In general, sharper signals are expected below the LCST and broader (or missing) signals after or close to the LCST, because liquid-state NMR spectroscopy is only expected to show signals related to sufficiently soluble/mobile polymers.^40^ Notably, we measured the LCST transitions in D_2_O. Thus, the values may be slightly different from those obtained in H_2_O. Previous studies have shown that the LCST of poly(NIPAm) in D_2_O is higher than in H_2_O by approximately 1 K.^41^

**Figure 2:**
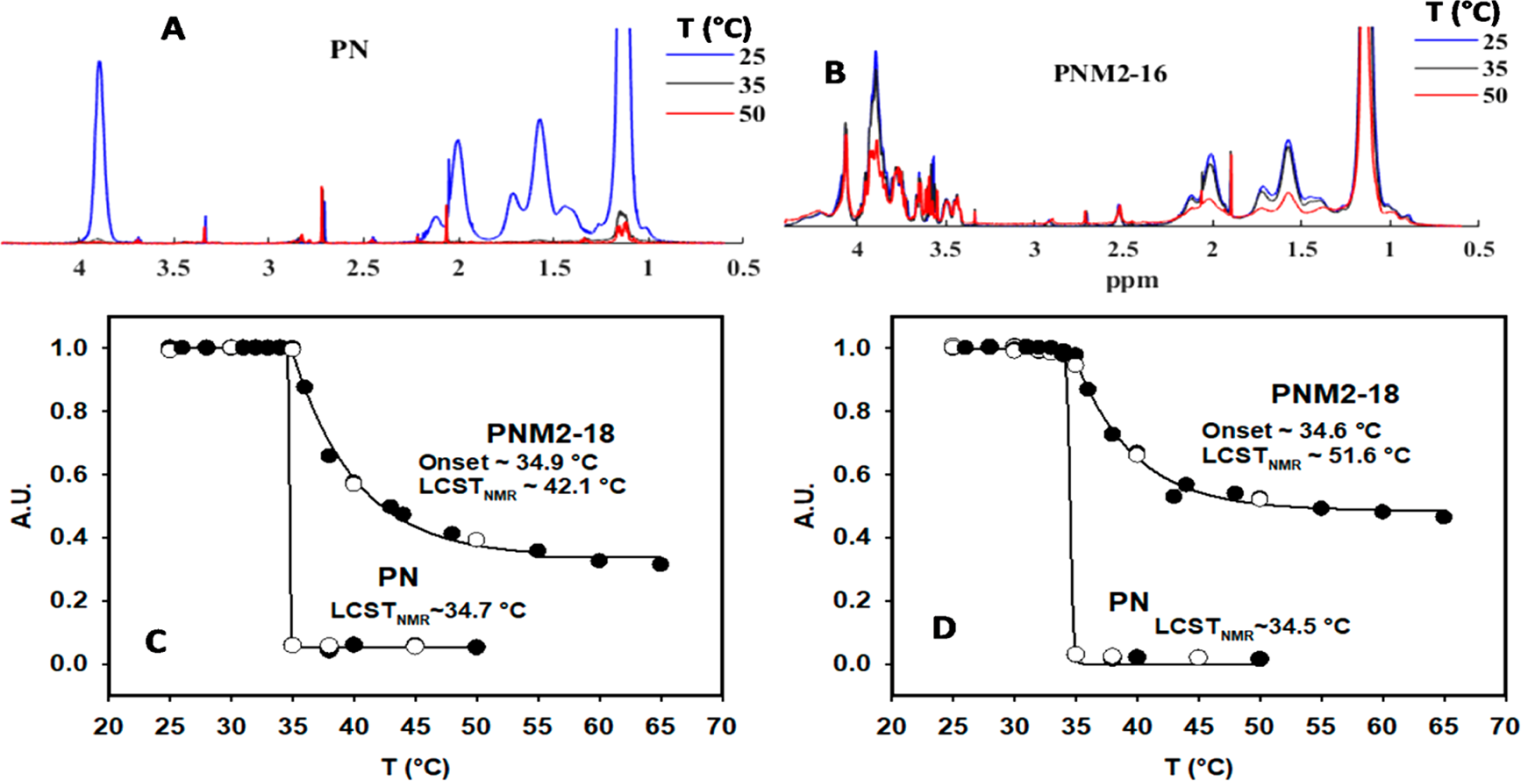
^1^H NMR (D_2_O, 500 MHz) spectra taken at 25, 35, and 50 °C for (A) PN and (B) PNM2–16. The decrease of signal intensity occurred gradually for PNM2–16 compared to PN. Determination of *T*_onset_ and LCST_NMR_ for PNM2–18 compared to PN: (C) from the chemical shift at 1.29–1.90 (H1, C**H**_2_) and (D) from the chemical shift at 3.79–4.12 (H5, N–C**H**(CH_3_)_2_). Open circles correspond to a cooling cycle and filled circles to a heating cycle.

Figure 2A shows the initial screening of the acquired ^1^H NMR spectra at 25, 35, and 50 °C for PN [poly(NIPAm)], as well as for the glycopolymer PNM2–16. As expected, the intensities of all signals from sample PN were practically zero (signal undetectable) at 35 °C due to the “coil-to-globule” transition that poly(NIPAm) underwent at the LCST (Figure 2A).^39,42^ In comparison, the glycopolymers, here illustrated by sample PNM2–16 (Figure 2B), showed that at 35 °C the intensity of the signals related to the poly(NIPAm) segments remained largely unchanged compared to the spectrum at 25 °C. Even at 50 °C, there was only a partial loss of intensity. This was expected because the poly(NIPAm) segments underwent a thermal random coil-to-globule transition upon heating, while the sugar segments remained solubilized. We have observed that the homopolymer of M_2_EMA did not undergo a thermal transition upon heating (data not shown). In other words, the sugar segments may have hampered the aggregation and precipitation of the copolymer. This behavior was modeled as previously described (see SI section C) to derive the parameters *T*_onset_ and LCST_NMR_ reported in Table 1.

Table 1 shows that the onset of the thermal transitions (*T*_onset_) was similar for all synthesized polymers and occurred at the LCST for poly(NIPAm) of ∼35 °C. As expected, *T*_onset_ was equal to LCST_NMR_ for sample PN, since the thermal transition from coil to globule was sharp. In comparison, the glycopolymers showed LCST_NMR_ above *T*_*o*nset_ due to a gradual thermal transition as previously described. In terms of the effect of the degree of substitution of sugar units and their length on the LCST transitions, we observed that the glycopolymers of M_2_EMA had statistically significantly higher LCST_NMR_ values compared to the ones based on M_1_EMA (Figure S3B in SI). In general, we observed that the higher the molar content of the M_*n*_EMAs, the lower the estimated LCST_NMR_. We will discuss possible reasons for this trend in section 3.5. Additionally, the reference copolymer based on HEMA (PNEMA-22) showed a significantly lower onset of thermal transition (30.8 ± 0.8 °C) compared to the rest of the samples. This difference was significant and independent of the purity of the reference sample (PNEMA-22 or PNEMAP-22). The effect of copolymerization with HEMA was small on the thermal transitions of poly(NIPAm) compared to the effect induced by the glycomonomers. This shows that the effect on the thermal transition of the poly[NIPAm] parts in the glycopolymers was most likely due to the introduction of sugar moieties, not only to the acrylate parts. Figure 2C,D also shows that the spectra taken during the heating cycle (filled circles) and during the cooling cycle (empty circles) were very similar (negligible hysteresis). This verified the reliability of the method used to estimate polymer segment mobility from the spectra and to demonstrate that the changes were reversible.

We want to point out that there was a difference in the estimation of LCST_NMR_ calculated from the chemical shifts corresponding to protons in the main chain of the poly(NIPAm) segments (H1 and H2 in Figure 1) compared to the estimation from the chemical shifts of the protons in the side chain (H5, H6, and H7 in Figure 1). In general, the loss of intensity from the main chain protons due to temperature increase occurred at a faster rate than for the side chain protons. This was indicative of a difference of mobility of the poly(NIPAm) segments of the polymer backbone compared to the side chains. This difference in mobility has been systematically studied by Futscher and co-workers.^43^ They studied the molecular changes in poly(NIPAm) and NIPAm in solution across the LCST transition, and determined that the hydrogen bonding with the amide groups in the side chains is different from the hydration of the hydrophobic main chain hydrocarbons.^43^ However, in the present study the signals used for the side chain calculations, illustrated in Figure 2D for proton H5, overlapped with signals corresponding to the glycopolymer segments. Consequently, we reported the calculated *T*_onset_ and LCST_NMR_ for the main chain, illustrated for H1 in Figure 2C.

We did not investigate the role of the molecular weight in the present study. However, the data in Table 1 shows that the parameter *T*_onset_ is not significantly different for the series of glycopolymers and reference sample PN. This indicated that within the molecular weight range of the samples investigated, no significant effect of the temperature at which the thermal transition started was observed. Still, the role of the molecular weight on the thermoresponsive behavior beyond *T*_onset_ was not studied systematically. This requires substantial effort in terms of synthesis and purification, beyond the scope of the present study. The determination of *M*_w_ of poly(NIPAm) homopolymers and copolymers by SEC is challenging, and the validity of these measurements have been discussed elsewhere.^44,45^ For example, Furyk and co-workers report significant differences between the *M*_w_ value of finely fractionated poly(NIPAm) samples estimated with SEC using THF as the mobile phase and the corresponding value determined by DLS in methanol. A recent study reported that SEC using methanol as a mobile phase is a suitable system for *M*_w_ determination of poly(NIPAm).^44^

DLS, cryo-TEM, and SAXS techniques were employed to obtain further knowledge on the structural changes associated with the thermal transitions observed by NMR spectroscopy. While NMR data provides information about the immediate vicinity of the atoms, DLS and SAXS reveal information on how this correlates with the polymer structural changes and aggregation, while cryo-TEM provides images of the structure and morphology of polymers/polymer aggregates. Hence, these techniques provide complementary information.

**Figure 3:**
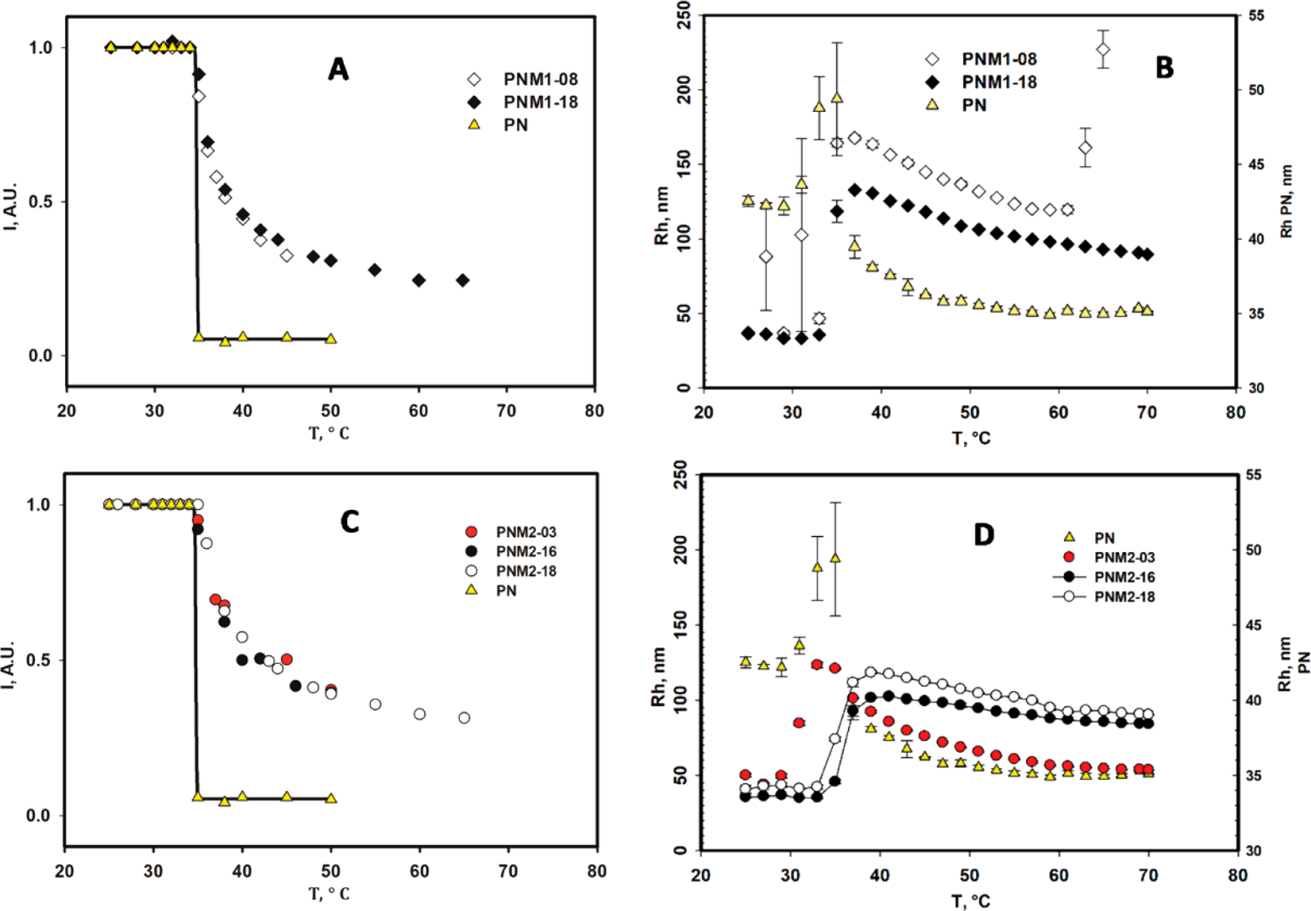
Thermal transitions of the glycopolymers and poly(NIPAm) (PN) studied by NMR spectroscopy (A and C) and by DLS (C and D).

Comparison of the NMR data and the results from the DLS analysis is shown in Figure 3. Parts A and C summarize the thermoresponsive behavior of poly(NIPAm*-co-*M_1_EMA)s and poly(NIPAm*-co-*M_2_EMA)s, respectively, as revealed by the intensity of the signal at 1.29–1.90 (H1, CH_2_) by NMR. Parts B and D show the corresponding change in hydrodynamic radius (*R*_h_) measured by DLS from 25 to 70 °C. The NIPAM homopolymer sample PN has been included for comparison.

Figure 3 shows that DLS and NMR spectroscopy data was temperature independent in the range 25–35 °C. According to DLS, the *R*_h_ reached a maximum size around ∼37–39 °C, followed by gradual shrinking up to 70 °C. Therefore, the results will be discussed in terms of these two temperature ranges in the following sections. We note here that the increase of *R*_h_ in the DLS data is expected, as the conformational transition of the polymer led to a decrease of solubility and hence aggregation.

Notably, while all samples showed the same trend in a given temperature range, sample PNM2–03 showed a shift toward lower temperatures in DLS (Figure 3D). PNM2–03 has an especially low sugar content (3 mol %). This sample illustrated that a certain degree of substitution of sugar is required to induce an effect on the thermal transition of poly(NIPAm). That is, at this low degree of sugar substitution the increase of *R*_h_ started earlier than for glycopolymers with at least 8 mol % sugar content (PNM1–08), and the shrinkage of the *R*_h_ after 35 °C was much more pronounced than for the rest of the samples. This was an indication that there were not enough sugar moieties to stabilize all of the poly(NIPAm) segments. Hence, the thermal transition was only slightly influenced by the hydrophilic interaction of the few sugar units. These results suggested that in the present case the sugar content had to reach a value between 3 and 8 mol % to induce an effect on the LCST of poly(NIPAm). Consequently, we then mostly focused on studying samples with content above 8 mol % by SAXS and cryo-TEM.

The ^1^H NMR data for PN (∼3 mg/mL) showed a very clear sharp transition at 34.7 °C. This was in agreement with the DLS data that showed a sharp increase of *R*_h_ at ∼35 **°**C, indicating the formation of intermolecular aggregates as a consequence of conformational changes of the polymer leading to the decrease of solubility. Large aggregates eventually sediment; however, a certain amount of polymer remains, leading to a decrease of the average *R*_h_ as monitored by DLS. The remaining particles, so-called mesoglobules, have been widely reported in the literature in relation to PolyNIPAm.^46^ Additionally, we confirmed that a solution of PN (∼30 mg/mL) becomes turbid when submerged in a water bath at 35 °C (Figure S7). This was not observed for the glycopolymer samples.

### 3.3 Thermoresponsive Characterization of Poly(NIPAm*-co-*M_*n*_EMA)s at 25–35 °C

**Figure 4:**
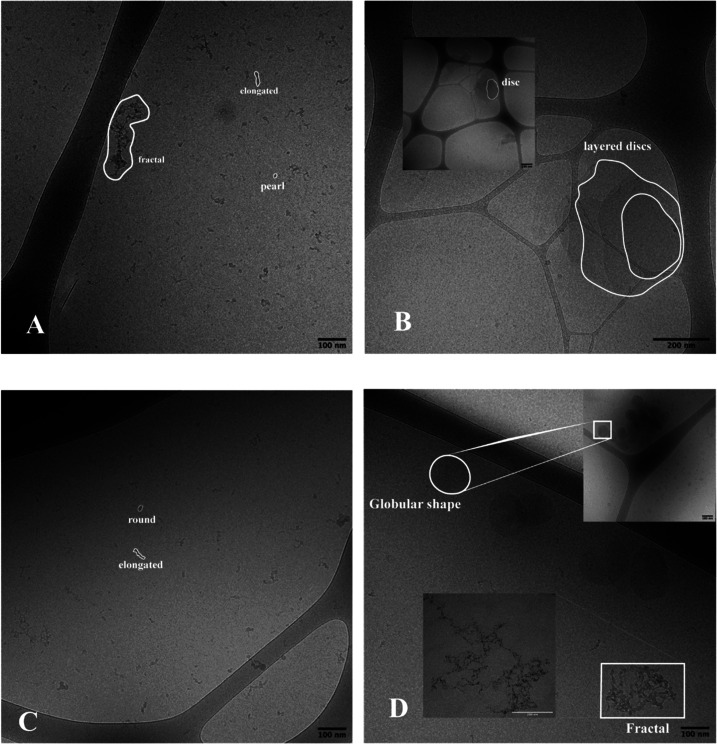
Cryo-TEM images prior to blotting at 10-fold dilution (∼1 mg/mL) of PNM1–08 incubated at 25 °C (A) and at 50 °C (B), and of PNM2–16 incubated at 25 °C (C) and at 50 °C (D). Structures observed: (1) pearl-shaped aggregates, (2) elongated structures; (3) disc-shaped structures that overlap; (4) globular structures; (5) fractal aggregates.

As previously mentioned, DLS and NMR spectroscopy data were temperature-independent in the range 25–35 °C. In this range, the hydrodynamic radius (*R*_h_) was between 35 and 62 nm. Additionally, cryo-TEM images of PNM1–08 and PNM2–16 at 25 °C (Figure 4A and C, respectively) already showed a large amount of small aggregates, 20–40 nm in diameter, and elongated structures of up to 80 nm. These dimensions suggested that the polymers had already aggregated at 25 °C into pearl-shaped objects. We found the same type and size of aggregates in all samples at 25 °C regardless of the type, size, or length of the sugar substituents. The aggregation occurred without any external trigger and was likely driven partly by attractive interactions between the sugar moieties, since poly(NIPAm) segments would not undergo any conformational change below ∼35 °C, as shown in Table 1. Furthermore, it has been reported that poly(NIPAm) exists in an expanded conformation in water below the LCST.^47^ Hydrogen bonds are formed between some sugars. In particular, Abeyratne-Perera and Chandran have demonstrated that mannose surfaces self-latch via hydrogen bonding.^48^ Similar self-assembly without external stimuli has been observed in double-hydrophilic block glycopolymers (DHBGs) of poly(ethylene) glycol and a polymannose in which the polymer could self-assemble into spherical structures through hydrogen bonding.^49^ Another example of such self-assembly has been reported for DHBGs of poly(2-hydroxyethyl methacrylate)*-b-*poly(2-(β-glucosyloxy) ethyl methacrylate) [PHEMA*-co-*PGEMA] in milli-Q water.^4^ These glucose-based block copolymers showed *R*_h_ from 4.25 to 19.8 nm depending on the length of the PHEMA block, according to DLS at room temperature.^4^ The authors proposed that the glycopolymers self-assemble into micellar structures. Here, given that we have glycopolymers with random monomer distribution, the self-assembled structures are expected to be heterogeneous. This was confirmed by calculating the shape parameter ρ = *R*_g_/*R*_h_ from the DLS data at 25 °C for glycopolymer PNM2–16 (Table S2). In comparison, a solid sphere gives ρ ∼ 0.775.^50^ For PNM2–16 at 25 °C, ρ was equal to 1.22, corresponding to an elongated shape which confirms the lack of homogeneous structures. Nevertheless, this type of self-aggregation of synthetic glycopolymers is highly desirable, as it does not rely solely on hydrophobic interactions.

**Figure 5:**
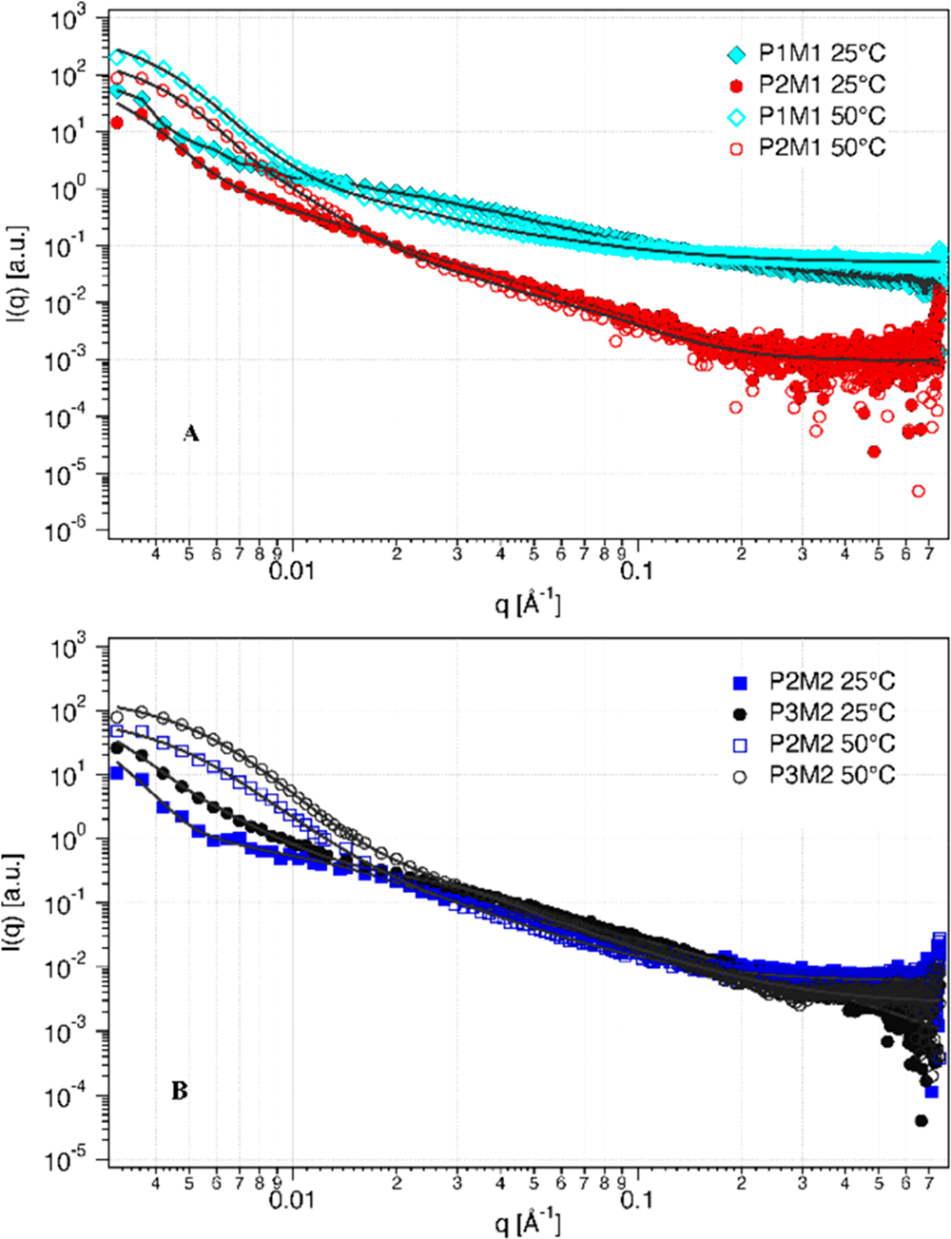
(A) SAXS curves of PNM1–08 (light blue rhombus) and PNM1–18 (red circles) (10 mg/mL) at 25 °C (filled symbols) and 50 °C (empty symbols). Solid lines are fits to the corrected Beaucage model. (B) SAXS curves of the PNM2–16 (dark blue square) and PNM2–18 (black circles) (10 mg/mL) at 25 °C (filled symbols) and 50 °C (empty symbols). Solid lines are fits to the corrected Beaucage model and polymer micelle model (for PNM2–16 and PNM2–18).

Further characterization was done by SAXS. At 25 °C, the SAXS data showed that all of the samples exhibited similar scattering profiles in solution. Figure 5A,B shows a gradual change in the power-law decay through the extended *q*-range in the scattering curves at 25 °C. Such scattering behavior is consistent with a self-similarity in the morphology of the different polymers in solution and suggested fractal-like behavior. All of the curves at 25 °C shown in Figure 5 were fitted to the Beaucage model,^30,31^ and the obtained results are summarized in Table 3.

**Table 2:** Summary of Characteristic *R*_h_ Values Measured by DLS of Poly(NIPAm*-co-*M_*n*_EMA) Solutions^a^

	parameter			
sample designation	*R*_h_ @ 25 °C [nm]	*R*_h_ max^b^ [nm]	*R*_h_ @ 50 °C [nm]	*R*_h_ min^c^ [nm]
PNM1–08	36 ± 0.9	168 ± 1.8	137 ± 0.8	119 ± 0.9
PNM1–18	37 ± 0.9	133 ± 0.9	107 ± 0.8	90 ± 0.4
PNM2–03	50 ± 1.4	123 ± 1.9	69 ± 0.4	54 ± 0.1
PNM2–16	36 ± 0.2	102 ± 0.5	95 ± 1.1	84 ± 0.7
PNM2–18	41 ± 2.7	118 ± 0.8	106 ± 0.6	90 ± 0.1
PN	42.5 ± 0.23	49.4 ± 3.8	n.a.	n.a.
PNEMAP-22	16.5 ± 0.8	335.8 ± 1.9	300 ± 2.6	n.a.

a *R*_h_ values reported for each temperature correspond to the average of 3 measurements.

b Measured as the maximum *R*_h_ in the DLS curve.

c Measured as the minimum *R*_h_ reached in the DLS curve after 35 °C.

**Table 3:** Parameters Obtained from the Fitting Poly-(NIPAm-*co*-M_*n*_EMA) SAXS Data at 25 °C to the Corrected Beaucage Model for a Fractal Object^a^

	parameters (fractal object)					
sample designation^b^	*R*_g_ [nm]	*d*	*R*_sub_ [nm]	*L*_p_ [nm]	*d*_sub_	χ^2^^b^
PNM1–08	89	3.70	14	24	1.52	7.33
PNM1–16	75	4.40	14	24	2.00	1.45
PNM2–16	85	3.3	12	21	1.54	1.94
PNM2–18	80	3.2	5	8	1.50	2.27

a Detailed description of the parameters can be found in Materials and Methods sections.

b Statistical parameter that quantifies the differences between an observed data set and an expected data set.

Table 3 shows that the radius of gyration (*R*_g_) of the glycopolymers at 25 °C was within 75–89 nm. This correlates to the cryo-TEM images showing the elongated shapes formed by the smaller pearl-shaped structures. The *R*_g_ at 25 °C decreased with increasing content of sugar monomers included in the structure regardless of the number of mannose units. This effect was more clearly seen from the SAXS data of the disubstituted glycopolymers. Hence, the magnitude of *R*_g_ decreased with the level of substitution, and so did the size of the subunit (Table 3, *R*_sub_), even if the difference in sugar content between the two disubstituted glycopolymers was narrower (16 vs 18 mol %) compared to the monosubstituted (8 vs 16 mol %). The persistence length (*L*_p_) that characterizes the chain stiffness remained constant for the monosubstituted glycopolymers (∼24 nm) and decreased significantly for the disubstituted ones (21 vs 8 nm).

It can be argued that for a larger density of bulky side groups, the chain flexibility would decrease, as has previously been observed with softwood hemicellulose with galactose side groups.^27^ However, in the case of hemicellulose the backbone is significantly more water-soluble than the poly(NIPAm) backbone. When temperature increases and there is a small change in solvent quality, hemicellulose would remain in solution, while poly(NIPAm) would precipitate. In our glycopolymers, the introduction of the sugar groups conferred even more solubility to the polymer backbone. Concurrently, the sugar moieties tended to show an attractive interaction, which was likely to be a combination of, e.g., hydrophobic interaction, specific orientation, and hydrogen bonding. The sugars interacted attractively with each other and led to a closer contact between the chain backbones as a “zip lock”, thus forming aggregates even at 25 °C without precipitation. Therefore, we concluded that the glycopolymers can aggregate below the LCST of poly(NIPAm) because of a combination of different effects partly induced by the presence of the sugar moieties. This behavior may be advantageous in applications where self-assembly is required.

### 3.4 Temperature Dependent Conformational Changes of Poly(NIPAm*-co-*M_*n*_EMA)s above 35 °C

We studied the glycopolymers before reaching the LCST of poly(NIPAm) (estimated at ∼35 °C), concluding that there was some form of aggregation partly driven by attractive interaction. This is likely due to a combination of, e.g., hydrophobic interaction and specific orientation between the sugar moieties and was therefore temperature-independent. Above 35 °C, however, we expected to see structural changes driven by the segments of poly(NIPAm).

After the temperature rose beyond 35 °C, DLS data showed a rapid increase of *R*_h_, followed up by a steady decrease of *R*_h_ up to 70 °C for all samples except PNM1–08, for which this decrease was up to 63 °C (this will be discussed in detail below). The initial increase in *R*_h_ was in the order of 2–4 times the average *R*_h_ at 25 °C, reaching a semiplateau in 102–168 nm at about ∼37–39 °C (reported as *R*_h_ max in Table 2). This behavior was modeled with a linear regression which revealed that this increase in *R*_h_ was faster in poly(NIPAm*-co-*M_1_EMA)s compared to poly(NIPAm*-co-*M_2_EMA)s. The initial increase of *R*_h_ was due to unfavorable poly(NIPAm)–water interactions that led to the collapse of the polymer chains, and eventually to aggregation due to hydrophobic interaction among the collapsed chains.^51^ At the same time, the sugar–sugar interactions were still present to interfere with the hydrophobic interactions. Even if in all cases the thermal transition started at similar temperature, this caused a gradual thermal transition, in contrast to a sharp transition as previously described.

As the temperature increased beyond ∼39 °C, we observed a gradual decrease of *R*_h_ in the DLS data (Figure 2) over a wide range of temperatures. The minimum size reached is reported as *R*_h_min in Table 2. This decrease of *R*_h_ was first linear and then proceeded at a slower rate (second degree polynomial fit). The gradual decrease in *R*_h_ suggested that the polymer coils would shrink in size with increasing temperature. A similar decrease of *R*_g_ after an increase has also been seen in double-hydrophilic thermoresponsive block glycopolymers (DHTBG) of poly(di[ethylene glycol]methyl ether methacrylate) and a galactose functionalized poly(6-*O*-vinyladipoyl-d-galactose).^16^ It was suggested that this shrinking behavior was most likely related to the increase in hydrophobicity of the hydrophobic segments leading to a shrinkage of the structures present.^16^ Accordingly, cryo-TEM images recorded at 50 °C showed that the small aggregates observed at 25 °C appear to have rearranged into large irregular structures. These structures resembled disk-like aggregates for samples with M_1_EMA and globular aggregates for samples with M_2_EMA (Figure 4). These aggregates were sometimes attached together to form fractal aggregates. In particular, we calculated the shape parameter ρ = *R*_g_/*R*_h_ to be 0.85 from DLS data collected at 45 °C for PNM2–16 (Table S2). This suggested a somewhat hollow spherical shape (*R*_g_*/R*_h_ ∼ 1 is indication of a hollow sphere)^16^ which correlates with the globular aggregates seen by cryo-TEM. The disc-like aggregates showed a lower contrast against the background than the globular type of aggregates. The exact organization within the discs was challenging to determine within the scope of the present study. It is, however, tempting to assume that these type of structures may be regarded as a lamellar type of structure, as observed in some block copolymer systems.^52^ Additionally, along the globular/disc shapes we saw another type of fractal aggregates apparently made up of the elongated aggregates seen at 25 °C (Figure 4). The formation of aggregates was due to unfavorable poly(NIPAm)–water interactions that led to the collapse of the polymer chains. However, further aggregation and eventual precipitation was hampered due to the presence of the sugar moieties that rendered the polymer slightly more hydrophilic. This behavior can be likened to coacervate-forming polymers.^51^ These polymers cannot cause enough dehydration of their chains above the LCST, but instead, they form micrometer-scale dispersions and cannot undergo a drastic conformational change. It has been reported that this type of polymer is better suited for biological applications, because their gentle thermoresponsive behavior is less disruptive in biological or biomimetic systems.^51^ When poly(NIPAm) aggregates and precipitates, it is due to a gain in entropy because of the dehydration of the hydrophobic moieties of the polymer, which in turn compensates for the loss of entropy arising from the collapse of the polymer chains into globules.^51^ By introducing sugar moieties as pendant groups, it is likely that the gain of entropy decreased and the chains could not undergo full dehydration.

We also studied the glycopolymers by SAXS at 50 °C, as a representative temperature in the temperature range in which the *R*_h_ was gradually decreasing. The results are summarized in Table 4. Compared to their size at 25 °C, the size of the polymer particles (*R*_g_) at 50 °C in general decreased by about 26–36% (one sugar) and by 45–54% (two sugars) depending on the degree of sugar substitution, as compared to their size at 25 °C. This decrease was also seen in the size of the subunit (*R*_sub_) indicating that the whole structure was collapsing. This correlated with the decrease in size observed by DLS.

**Table 4:** Parameters Obtained from Fitting Poly-(NIPAm*-co-*M_*n*_EMA) SAXS Data at 50 °C to the Corrected Beaucage Model and Polymer Micelle Core–Corona Model^a^

	parameters											
	fractal model						micelle model					
sample designation	*R*_g_ [nm]	*d*	*R*_sub_ [nm]	*L*_p_ [nm]	*d*_sub_	χ^2^	*R*_g_ core [nm]	*R*_g_ corona [nm]	*N*_head_	*V*_head_ [nm^3^]	*V*_tail_ [nm^3^]	χ^2^^b^
PNM1–08	57	4.7	8	14	1.6	11.68	-	-	-	-	-	
PNM1–16	55	3.90	5	9	2.90	1.57	-	-	-	-	-	
PNM2–16	47	4.65	8	15	2.27	2.04	16	28	30	22	322	1.76
PNM2–18	37	3.42	3	5	2.15	2.16	14	22	41	7	100	1.99

a Detailed description of the reported parameters can be found in Materials and Methods sections. All values are averages from three measurements.

b Statistical parameter that quantifies the differences between an observed data set and an expected data set.

Figure 5 shows that the SAXS data of the glycopolymers recorded at 50 °C exhibited a similar trend as the data recorded at 25 °C, but with a more pronounced increase in the intensity with *q* at the transition from an intermediate- to a low-*q* region. The large exponent at low-*q* can be described as Porod behavior (∼*q*^–4^) indicating that polymers collapsed into compact objects with sharp interfaces.^53^ This type of increase in the power-law behavior with temperature has been previously reported in the literature for temperature-responsive triblock copolymers that contain poly(NIPAm) blocks.^29,30^ These compact objects corresponded to those described as disc-shaped structures on the basis of the cryo-TEM results (see Figure 4B) for monomannose substituted glycopolymers and globular-shaped structures with sharp edges for the disubstituted ones (Figure 4D). As previously described, we also observed by cryo-TEM irregular fractal aggregates that coexisted with the globular/discs aggregates. SAXS data showed that the scattering was dominated by the former, as indicated by the *d*_sub_ ≈ 2 at the intermediate *q*-range characteristic to dense mass fractals.^31^ Hence, the data was fitted using the same model (fractal) as employed for the data at 25 °C (Table 4). However, it was also possible to fit the SAXS data at 50 °C for the dimannose-substituted glycopolymers (PNM2–16 and PNM2–18) to a polymer micelle core–shell model, i.e., a spherical particle with a dense core consisting of polymer heads and a corona consisting of Gaussian polymer tails.^54^ This model is often employed to describe block copolymer micelles.^55^ Here, we made an assumption that the globular structures seen in cryo-TEM were micelles with a core made of poly(NIPAm) with a scattering length density (SLD) of 0.1 × 10^–6^ Å^–2^ and that the corona consisted of mannan with an SLD of 0.145 × 10^–6^ Å^–2^. According to the fit shown in Table 4, the glycopolymers with two mannose units formed “micelle-type” particles with an overall radius of 44 and 36 nm for PNM2–16 and PNM2–18, respectively (Table 4). Particles with a higher substitution degree of dimannose (18 mol %) had a slightly smaller “core” but a “corona” with a larger radius than the less substituted ones (16 mol %). This suggested that a smaller amount of collapsed chains was surrounded by a larger amount of sugar units “protecting” the core. Here, the water could interact with the sugar units preventing further aggregation and consequent precipitation. This is expected to give a larger colloidal stability of these polymers at higher temperature. We acknowledge that SAXS data are easier to interpret for homogeneous samples. However, we have applied here one of the most accepted approaches for analysis of SAXS data for polymer systems.^29−31^ We also note that with SAXS we probe structures at a smaller length scale than DLS and cryo-TEM; hence, it complements the characterization of challenging samples.

### 3.5 Aggregation and Precipitation of PNM1–08

As we previously described, DLS, NMR, and SAXS data showed that the structures formed upon heating beyond the LCST of poly(NIPAm) did not reach a plateau value with increasing temperature, but continued to shrink up to 70 °C. However, it is expected that the structures would eventually aggregate and collapse, given that the LCST behavior is entropy driven. This assumption was validated using sample PNM1–08, for which DLS showed that at 63 °C there was a sharp increase of *R*_h_, which indicated further aggregation and eventually the copolymer precipitated. This was also observed by NMR spectroscopy. At temperatures beyond 60 °C, it was not possible to record any spectra because there were no phases with sufficient mobility. Thus, the shimming of the sample failed. It is expected that all the other samples would eventually also aggregate and precipitate at temperatures above 70 °C. Sample PNM1–08 was based on M_1_EMA and had a low mannose unit content compared to the other glycopolymers. A higher sugar content and long sugar units prevented the aggregation of collapsed chains up to 70 °C. These observations were in agreement with that seen for poly(NIPAm) based glycopolymers with α-linked mannose units, synthesized by Paul and co-workers.^17^ They observed that glycopolymers functionalized to a high mannose content (34 and 97 mol %) did not show a temperature responsive behavior up to 40 °C, in contrast to samples with sugar contents under 7 mol %.^17^ Hence, the thermal responsiveness is affected by the sequence of the sugar units within the polymer backbone. The degree of dehydration will depend on the sugar unit distribution and their hydrophilicity would govern the solubility of the polymer even above LCST of poly(NIPAm). In our case, given that the polymers were synthesized by free radical polymerization, it is not possible to study in detail how the sugar units are distributed along the polymer backbone. Hence, we cannot exclude that this distribution to some extent contributes to the trends seen in LCST_NMR_.

### 3.6 Conformational Changes as a Function of the Concentration

We believe that the type of structures formed as temperature increases were concentration dependent. For example, we imaged by cryo-TEM one sample of dimannose-substituted copolymer (PNM2–16) at the initial concentration and at 10-fold dilution (∼10 mg/L vs ∼1 mg/L) at 25 and 50 °C, respectively. At both temperatures, we observed the same type of structures as previously described. However, a larger amount of elongated particles and even fractal aggregates were seen at the higher concentration at 25 °C compared to the diluted sample (Figure S4). Similarly, at 50 °C, the effect of concentration was much more pronounced than at 25 °C. While both the diluted and the concentrated samples contained globular aggregates and fractal aggregates of similar dimensions, the former were found in higher numbers under dilute conditions, whereas the latter dominated under concentrated conditions (Figure S5). The polymers assembled randomly into mostly fractal-like structures at a high concentration, as expected for a strongly attractive interaction. Consistently, in a dilute sample more time was given for the polymer to aggregate in a more controlled and organized manner giving less extended aggregates.

## 4 Conclusions

We have synthesized biobased thermoresponsive glycopolymers from glycomonomers, prepared by enzymatic catalyzed synthesis, and NIPAm. The conventional free radical polymerizations reached high conversions already after 2 h. We then systematically characterized their solution properties and their conformational changes upon heating by employing a combination of ^1^H NMR, DLS, SAXS, and cryo-TEM. The glycopolymers were observed to aggregate at room temperature, partly due to the attractive interaction of the sugar moieties because of hydrophobic interactions, specific orientation, and hydrogen bonding. This behavior may be advantageous in applications where self-assembly is required. We then showed that the glycopolymers had an LCST-type phase transition, as well as aggregation properties beyond the LCST of poly(NIPAm).

We observed that upon increasing the temperature beyond the LCST of poly(NIPAm), the glycopolymers were able to rearrange into sharp-edged structures with various shapes (fractal, discs, and globular). The size and shape of these structures varied as a function of the size and degree of substitution of the mannose pendant moieties, offering handles for the variation of the structures to target specific applications. To the best of our knowledge, there is little research into the thermal transitions of thermoresponsive glycopolymers with a random distribution of sugar moieties along the polymer backbone, in particular, on glycopolymers featuring β-linked mannose units. We expect that the findings of this work will form the basis for the synthesis of a library of glycopolymers with diverse structures/function using alternative comonomers and/or other polymerization techniques. Although the amount of monomers available for the synthesis of these thermoresponsive glycopolymers was limited, the results from the systematic characterization study carried out here are promising. This certainly motivates further studies with respect their self-assembly behavior and thermally trigged response, as well as investigations on the possibility to trigger the affinity to sugar-binding biomacromolecules. Such studies will explore the potential biomedical and bioanalytical applications.

## Supporting Information Available

Expanded description of polymerization method, acquisition and processing of NMR data spectroscopy, procedure to calculate parameters *T*_onset_ and LCST_NMR_ reported in Table 1, additional DLS data, ^1^H and ^13^C NMR spectra, complete assignment of ^1^H and ^13^C NMR spectra, additional graphs for Table 1, and as well as additional cryo-TEM images and inserts of the SEC chromatograms, images of the LCST behavior of poly(NIPAm) and polymerization kinetic analysis for samples PNM1–18 and PNM2–18 (PDF)

## Abbreviations

Cryo-TEM: cryogenic transmission electron microscopy
DLS: dynamic light scattering
HEMA: 2-hydroxy ethyl methacrylate
LCST: lower critical solution temperature
M_*n*_EMAs: 2-(β-manno[oligos]yloxy) ethyl methacrylates)
M_1_EMA: 2-(β-mannosyloxy) ethyl methacrylate)
M_2_EMA: 2-(β-manno[bio]syloxy) ethyl methacrylate)
NMR: nuclear magnetic spectroscopy
poly(NIPAm*-co-*M_*n*_EMA)s: poly(*N*-isopropylacrylamide)-*co*-(2-[β-manno[oligo]syloxy] ethyl methacrylate)s
poly(NIPAm): poly(*N*-isopropylacrylamide)
SEC: size exclusion chromatography
SAXS: small-angle X-ray scattering
