# Supplementary material for: Thermoresponsive Glycopolymers Based on Enzymatically Synthesized Oligo-β-Mannosyl Ethyl Methacrylates and N-Isopropylacrylamide
Source: Biomacromolecules. 2021 May 7;22(6):2338–51. doi: 10.1021/acs.biomac.0c01615 (PMC8382249; doi:10.1021/acs.biomac.0c01615)
Supplement: Supplementary file 1 — bm0c01615_si_001.pdf [file bm0c01615_si_001.pdf]

**Supporting Information**

# Thermoresponsive Glycopolymers Based on Enzymatically Synthesized Oligo-β-Mannosyl Ethyl Methacrylates and *N*-Isopropylacrylamide

**Monica Arcos-Hernandez[a]*, Polina Naidjonoka[b‡], Samuel J. Butler[c‡], Tommy Nylander[b], Henrik Stålbrand[c]*, Patric Jannasch[a]***

*[a]Centre for Analysis and Synthesis, Department of Chemistry, Lund University, PO Box 124, S-221 00 Lund, Sweden*

*[b]Physical Chemistry, Department of Chemistry, Lund University, PO Box 124, S-221 00 Lund, Lund, Sweden.*

*[c]Department of Biochemistry and Structural Biology, Department of Chemistry, Lund University, PO Box 124, S-221 00 Lund, Sweden.*

## A. Detailed synthesis of poly(N-isopropyl acrylamide)-*co*-(2-[β-manno[oligo]syloxy] ethyl methacrylate)s [Poly(NIPAm-*co*-$M_nEMA$)s]

A total of seven different polymers were prepared in solution. We will refer to the glycopolymers as PNMX-YY where X is 1 or 2 for $M_1EMA$ and $M_2EMA$, respectively, and YY is the molar percentage of the glycomonomer after polymerization, as determined with NMR spectroscopy. Two glycopolymers (PNM1-08- and PNM1-18) based on $M_1EMA$ and three (PNM2-03, PNM2-16 and PNM2-18) with $M_2EMA$ were prepared. Additionally, two reference samples were prepared, a homopolymer of NIPAm (PN) and a copolymer of NIPAm and HEMA (PNEMA-18). Details of the experimental design of the polymerizations can be found in Table S1.

Copolymers of $M_nEMA$s and NIPAm and the homopolymer of NIPAm were synthesized via conventional radical polymerization in $D_2O$ at room temperature in an NMR tube, as previously reported.[1] A system of initiator potassium persulfate (KPS) and accelerator *N,N,N',N'*-tetramethylethylenediamine (TEMED) were used. The polymerizations solutions were designed with a monomer concentration of 250 mmol/L and monomer:initiator:accelerator ([M]:[I]:[A]) ratios of 100:0.4:0.6 unless otherwise stated (Table S1). The reference sample based on HEMA (PNEMA-18) was prepared in ethanol with 2,2'-azobis(isobutyronitrile) (AIBN) as initiator at 60 °C in a similar way as previously described.[2]

**Table S1.** Synthesis data of the thermoresponsive glycopolymers and reference polymers based on NIPAm.[a]

| Sample type | Designation | $y_{feed}$[b] $M_nEMA$ (orHEMA) [mol/mol] | [M] [mmol/L] | [M]:[I]:[A] | $M_nEMA$(or HEMA)/NIPAm[c] [mg/mg] |
|---|---|---|---|---|---|
| **Poly(NIPAm-*co*-$M_1EMA$)** | PNM1-08 | 0.07 | 250 | 100:0.4:0.6 | 0.21 |
| | PNM1-18 | 0.18 | 250 | 100:0.4:0.6 | 0.55 |
| **Poly(NIPAm-*co*-$M_2EMA$)** | PNM2-03 | 0.02 | 120[d] | 100:0.3:- | 0.10 |
| | PNM2-16 | 0.15 | 250 | 100:0.4:0.6 | 0.45 |
| | PNM2-18 | 0.18 | 250 | 100:0.4:0.6 | 0.86 |
| **Poly(NIPAm)** | PN | - | 250 | 100:0.4:0.6 | - |
| **Poly(NIPAm-*co*-HEMA)**[e] | PNEMA-22<br>PNEMAP-22 | 0.14 | 250 | 100:0.4:0.6 | 0.20 |

[a]I= initiator, KPS; A=accelerator, TEMED; M=monomer. PN and PNEMA-22 are reference materials; [b]$y_{feed}$ is the molar fraction of the comonomers polymerized with NIPAm (MnEMA or HEMA). [c]These values were estimated using data from $^1H$ NMR quantification (ERECTIC 2 method). [d]No accelerator was added in this synthesis and [M] is lower than the other syntheses. [e]PNEMA-22 and PNEMAP-22 were obtained from the same polymerization except PNEMA-22 is a crude sample and PNEMAP-22 was from the same polymerization solution but purified.

A volume of 250 mL of the enzymatic synthesis mixture was used for purification and isolation of the individual monomers, $M_nEMA$s, as previously described.[1] After purification the $M_nEMA$ monomer solutions contained 70-85% acetonitrile. The total mass available after downstream

processing was of 18.7 and 29.5 mg of $M_1EMA$ and $M_2EMA$, respectively. The reason for these small amounts was the low yield in the recovery and purification of the products. We are currently working to optimize the downstream processing steps to increase the recovery yield. Because of the small amounts of purified monomers available, we carried out the polymerizations at microscale in an NMR tube.

Before the polymerizations, a pre-determined volume of monomer solutions was subjected to acetonitrile evaporation in a rotary evaporator. A gradual exchange of water for $D_2O$ to the desired monomer concentration followed. This was done through careful evaporation of fractions of water at 37 °C in the rotary evaporator, adding $D_2O$ in multiple steps. The temperature was all the time kept below 37 °C (enzymatic reaction temperature) to prevent possible spontaneous polymerizations of the monomers. Once the desired volume was reached, the monomer solution was transferred to the NMR through a septum via a glass microscale syringe equipped with a long needle. NIPAm, initiator (KPS) and accelerator (TEMED) solutions in $D_2O$ were prepared and degassed separately before addition to the NMR tube containing the $M_nEMA$ solution. The NMR tube was then sealed and the polymerization was carried out at room temperature.

The microscale polymerizations were challenging, mainly because the glycomonomers were shown to precipitate from solution during the concentration step. This leads to losses of the glycomonomers along the process, thus limiting the net amount of the glycomonomers available for polymerization. Thus, it was challenging to keep the exact amounts of monomers in the polymerizations. Optimization of recovery and purification methods on a large scale are subject of ongoing work. Nevertheless, we purified enough glycomonomers to prepare several samples at a microscale. Utmost care was taken to maintain all parameters close to the nominal values of the experimental design. The concentration of reactants was estimated during the polymerization via NMR (average predicted error of 2% measured in 5 external standards, as described in materials and methods) and these are the ones that are reported in Tables S1 and 1.

All the glycopolymers and the PNIPAm were kept in solution in the NMR tubes after synthesis without any further processing before the characterization. Only part of the reference PNEMA-22 solution was used to obtain a purified polymer due to low monomer conversion (See Table 2). The purified sample PNEMA-22 is labeled as PNEMAP-22. To obtain sample PNEMAP-22, some of sample PNEMA-22 was precipitated twice in 10 times volume of diethyl ether, filtered and rinsed. The recovered white powder was vacuum dried to constant weight, and then suspended in 600 μL of $D_2O$ for characterization. Even though this sample is a reference sample and does not contain glycomonomer, we used it as a model compound to attempt to elucidate and separate the effects on the thermoresponsive behavior of PNIPAm (i) from the introduction of the acrylate moieties and (ii) from the introduced sugar moieties. We also used PNEMA-22 and PNEMAP-22 to investigate if the presence of unreacted monomer had any effect on the thermo-responsive behavior.

It is worth noting that in sample PNM2-03, the monomer molar concentration in the polymerization was lower than in the rest of the polymerizations. Given the low amounts of

available glycomonomers, this reaction was the first polymerization performed and was designed to minimize the amount of glycomonomer used. Initially, the monomer molar concentration in the reaction was 54 mmol/L. No polymerization occurred at this low concentration so we increased the molar monomer concentration to 120 mmol/L by carefully evaporating some of the water in a rotary evaporator (Table S1). Fresh initiator solution was also added and the polymerization then progressed at room temperature.

## B. Nuclear Magnetic Resonance (NMR) spectroscopy, detailed description

To acquire $^1$H NMR spectra for each sample the spin-lattice (T1) was determined from a series of spectra using the standard inversion recovery sequence varying the delay time from 0.001 to 5 s. A relaxation delay of 5 × T1 (for a 90 ° pulse) and an acquisition time of 3 × T2 were used for the acquisition of the spectra. The 90 ° pulse was calibrated using the *pulsecal* program from Topspin. The number of scans and the parameter D1 was adjusted for each sample. All spectra for determination of low critical solution temperature (LCST) transitions were shimmed in solvent suppression mode with digitization mode set to *baseopt*. Spectra calibration, base line correction and phase correction were done using Topspin. In-house generated Matlab scripts, as well as the freely available Matlab application RBNMR[3], were used for spectra analysis.

## C. Determination of parameters $T_{onset}$ and $LCST_{NMR}$ from $^1$H NMR data

To determine the LCST transitions from the acquired spectra, the intensities of selected chemical shifts at different temperatures were calibrated to the intensity at 25 °C and plotted against temperature. The intensity is calibrated to the value of the intensity of the reference spectrum. All intensities in the series of experiments are then scaled with the same factor. This allows to immediately compare the spectra within the series of experiments.

A 5-parameter sigmoidal curve (equation S1) was then fitted using Sigmaplot® V.12.0. The fit provides with 5 parameters (a-e). From this fitting two parameters were derived, the $T_{onset}$ of the LCST (determined as the temperature of greatest slope, $x_0$ in Equation S1) and a parameter that we have named $LCST_{NMR}$, which was obtained by resolving the temperature ($x$) for when the $y$=0.5 (the intensity is half of the initial intensity $y$=1):

$$y = y_0 + \frac{a}{\left[1+ e^{-\left(\frac{x-x_0}{b}\right)}\right]^c} \qquad \text{Equation S1}$$

## D. DLS and SLS for sample PNM2-16 to determine shape factor

DLS and SLS data was measured to derive the shape factor $\rho = R_g/R_h$ reported in table S2 for sample PNM2-16 at 25 and 45 °C. The obtained scattering intensity $I(q)$ was brought to an absolute scale

using: [T. Zemb and P. Lindner, Neutron, X-Rays and Light Scattering Methods Applied to Soft Condensed Matter, North Holland, 2002.]

$$I(q) = \frac{\Delta I(q)}{I_{ref}(q)} \left(\frac{n}{n_{ref}}\right)^2 RR_{ref} \qquad \text{Equation S2}$$

where $\Delta I(q)$ is the background scattering, $n$ is the refractive index of the solution, and $I_{\mathrm{ref}}$ $(q)$, $n_{\mathrm{ref}}$ and $RR_{ref}$ are the scattered intensity, refractive index, and Rayleigh ratio, of the reference (toluene), respectively; $q$ is the magnitude of the scattering vector:

$$q = \frac{4\pi n}{\lambda_0} sin\left(\frac{\theta}{2}\right) \qquad \text{Equation S3}$$

where $\lambda_0$, $n$ and $\theta$ are the laser wavelength, refractive index of the solution and scattering angle, respectively. The radius of gyration $R_{\mathrm{G}}$ was calculated using Zimm plot. The derived $R_{\mathrm{g}}$ and $R_{\mathrm{h}}$ were used to calculate shape factor.

**Table S2.** DLS data of sample PNM2-16 to determine the shape factor.

| **Temperature [°C]** | $\boldsymbol{R_{\mathrm{g}}}$ **[nm]** | $\boldsymbol{R_{\mathrm{h}}}$ **[nm]** | $\boldsymbol{\rho = R_{\mathrm{g}}/R_{\mathrm{h}}}$ |
|---|---|---|---|
| 25 | 49.1 | 40.11 | 1.22 |
| 45 | 50 | 58.63 | 0.85 |

## E. $^{1}$H and $^{13}$C NMR spectra for sample PNM2-18.

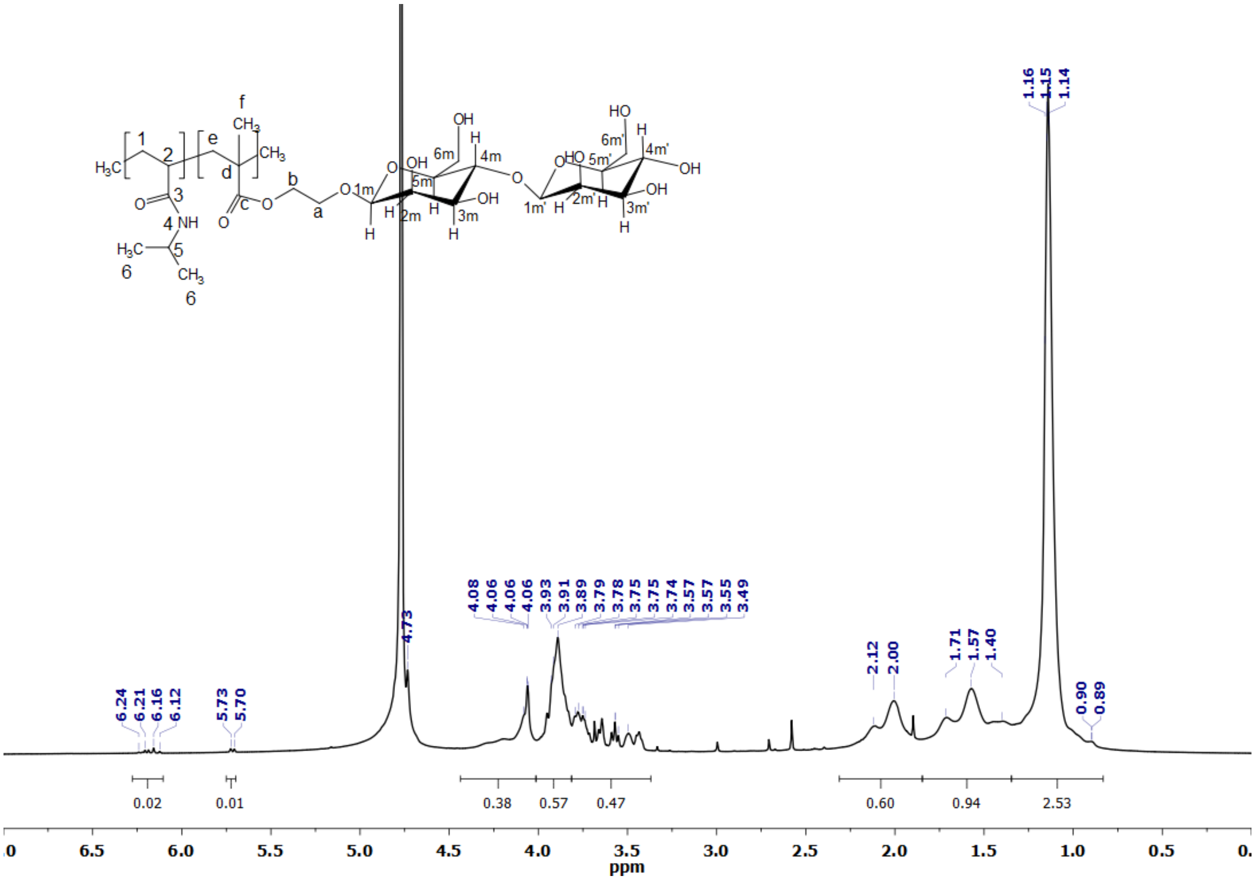


**Figure S1.** $^{1}$H NMR ($D_2O$, 500 MHz) spectra of poly(N-isopropylacrylamide-co-2-[β-manno(bio)syloxy] ethyl methacrylate) PNM2-18 taken at 25 °C chemical shift scale from residual HDO (δ 4.77) δ in ppm: 0.96–1.29 (m, H6+H7, N-C **H** ($CH_3$)$_2$), 1.29–1.90 (m, H1, C **H** $_2$ in main chain), 1.90–2.29 (m,H2, C **H** in main chain), 3.79–4.12 (m, H5, N-C **H** ($CH_3$)$_2$), 0.8 –1.2 (m, Hf, C **H**$_3$), 1.5 – 2.5 (m, He, C **H** $_2$), 4.74* (s, H1m, C **H**), 4.72* (s, H1m', C **H**), 4.44- 4.01 (m, C **H** $_2$ +C **H**, Hb+H2m'+H2m), 4.01-3.61 (m, C **H** $_2$ + C **H**, Ha + H6m'+H6m+H4m+H4m'+H3m'), 3.61-3.53 (m, C **H** , H4m), 3.42 (m, C **H** , H5m+H5m').

*Assigned in spectra at 45 ºC, HDO (δ 4.60)

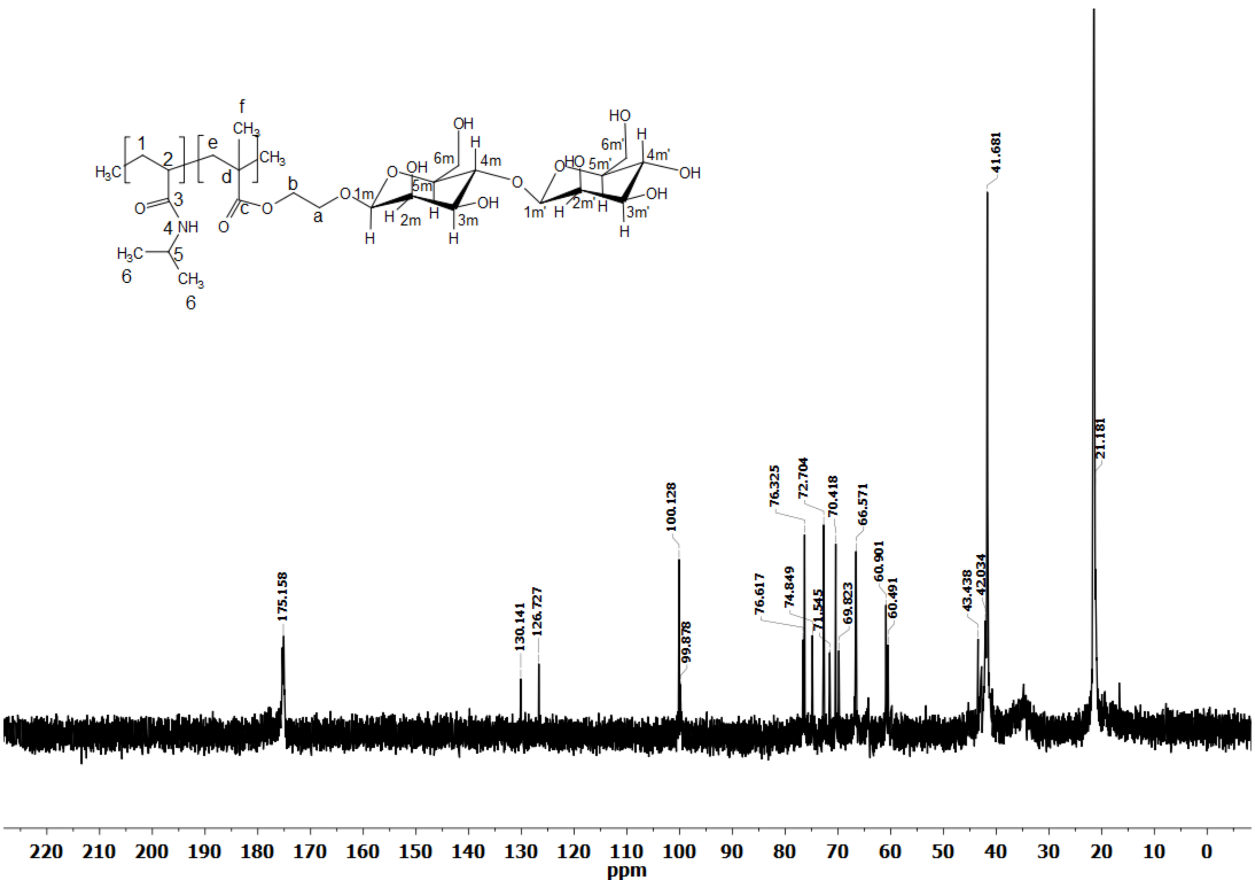


**Figure S2.** $^{13}$C NMR (D20, 125 MHz) spectra of poly(N-isopropylacrylamide-co-2-[β-manno(bio)syloxy] ethyl methacrylate) PNM2-18 taken at 25 °C δC in ppm, chemical shift scale from the unified scale according to IUPAC[4]: 175.2 (C3), 100.1 (C1m'), 99.9 (C1m), 76.6 (C4), 76.3 (C5m'), 74.8 (C5m), 72.8 (C3m'), 71.5 (C3m), 70.4 (C2m'), 69.8 (C2m), 66.6 (C4m'), 64.3 (Cb), 60.9 (C6m), 60.5 (C6m'), 41.7 (C2), 34.7 (C1), 21.5 (C6+C7)

## F. Detailed NMR peak assignments for all samples

**Poly(N-isopropylacrylamide-*co*-2-[β-mannosyloxy] ethyl methacrylate) [PNM1-08 and PNM1-18]**

$^{1}$H NMR ($D_2O$, 500 MHz) chemical shift scale from residual HDO (δ 4.77) δ in ppm: 4.72* (s, H1m, C **H**), 4.54-3.53 (m, br, Hb, C **H** $_2$; Ha, C **H** $_2$; H2m, C **H**; H5, N-C **H** $(CH_3)_2$; [H6m, H3m, H4m], C **H**), 3.32-3.42 (m, br, H5m), 1.29–2.35 (m, br, H1, C **H** $_2$ in main chain; He, C **H** $_2$; H2, C **H** in main chain), 0.8 –1.29 (m, br, Hf, C **H** $_3$; H6+H7, N-CH (C **H** $_3$)$_2$).
*Assigned in spectra at 45 °C, HDO (δ 4.60).
$^{13}$C NMR ($D_20$, 125 MHz) δC in ppm, chemical shift scale from the unified scale according to IUPAC[4]: 175.2 (C3), 99.9 (C1m), 76.3 (C5m), 73.0 (C3m), 70.5 (C2m), 66.7 (C4m), 64.4 (Cb), 61.0 (C6m), 41.7(C2), 34.7(C1), 21.22 (C6+C7)

**Poly(N-isopropylacrylamide-*co*-2-[β-manno(bio)syloxy] ethyl methacrylate) [PNM2-03, PNM2-16 and PNM2-18]**

$^{1}$H NMR ($D_2O$) chemical shift scale from residual HDO (δ 4.77) δ in ppm: 0.96–1.29 (m, H6+H7, N-C**H** $(CH_3)_2$), 1.29–1.90 (m, H1, C**H**$_2$ in main chain), 1.90–2.29 (m,H2, C**H** in main chain), 3.79–4.12 (m, H5, N-C**H** $(CH_3)_2$), 0.8 –1.2 (m, Hf, C**H**$_3$), 1.5 – 2.5 (m, He, C**H**$_2$), 4.74* (s, H1m, C**H**), 4.72* (s, H1m', C**H**), 4.44- 4.01 (m, C**H**$_2$ +C**H**, Hb+H2m'+H2m), 4.01-3.61 (m, C**H**$_2$ + C**H**, Ha + H6m'+H6m+H4m+H4m'+H3m'), 3.61-3.53 (m, C**H**, H4m), 3.42 (m, C**H**, H5m+H5m').

*Assigned in spectra at 45 °C, HDO (δ 4.60)

$^{13}$C NMR ($D_20$, 125 MHz) δC in ppm, chemical shift scale from the unified scale according to IUPAC[4]: 175.2 (C3), 100.1 (C1m'), 99.9 (C1m), 76.6 (C4), 76.3 (C5m'), 74.8 (C5m), 72.8 (C3m'), 71.5 (C3m), 70.4 (C2m'), 69.8 (C2m), 66.6 (C4m'), 64.3 (Cb), 60.9 (C6m), 60.5 (C6m'), 41.7 (C2), 34.7 (C1), 21.5 (C6+C7)

**Poly(N-isopropylacrylamide ) [PN]**

$^{1}$H NMR ($D_2O$) δ in ppm: 4.01-3.79 (s,br N-C**H** $(CH_3)_2$), 2.29-1.84 (m,br, C**H** in main chain), 1.84-1.30 (m, br, C**H**$_2$ in main chain), 1.30-0.92 (m, br, N-CH (C**H**$_3$)$_2$).

$^{13}$C NMR ($D_20$, 125 MHz) δC in ppm, chemical shift scale from the unified scale according to IUPAC[4]: 175.4 (C3), 42.0 (C2), 34.7 (C1), 21.80 (C6, C7).

## G. Analysis of LCST and $LCST_{NMR}$ by $^{1}$H NMR spectroscopy

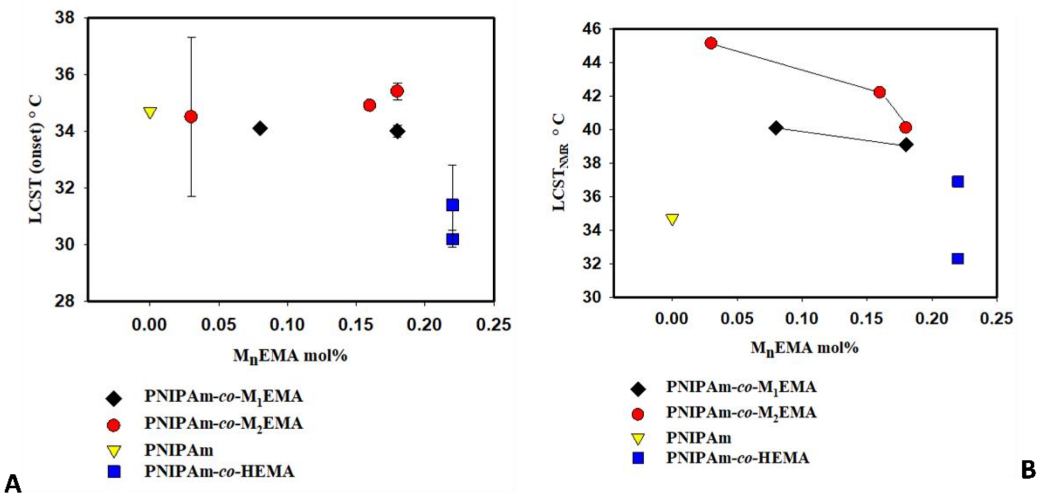


**Figure S3.** Estimated $T_{onset}$ of the transition (A) and $LCST_{NMR}$ (B) of the glycopolymers and the reference materials by NMR analysis. Error bars correspond to the SSE of the curve fitting for the respective parameter.

## H. Cryo-TEM concentration dependence for sample PNM2-16

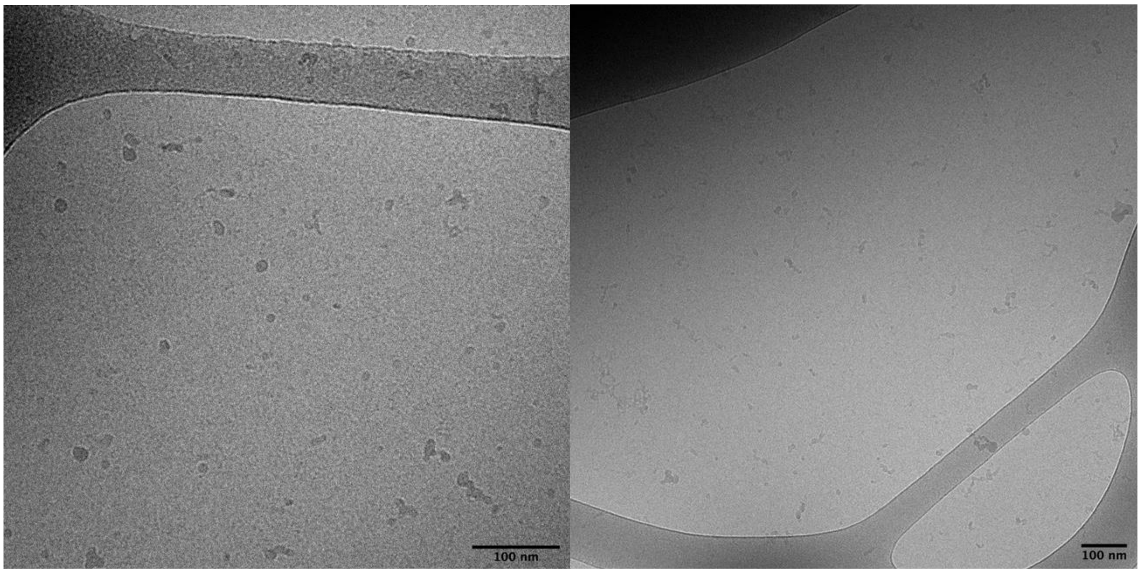


**Figure S4.** Cryo-TEM images of PNM2-16 incubated at 25 °C prior to blotting at 10-fold dilution ~1 mg/mL (left) and at the initial concentration ~10 mg/mL (right).

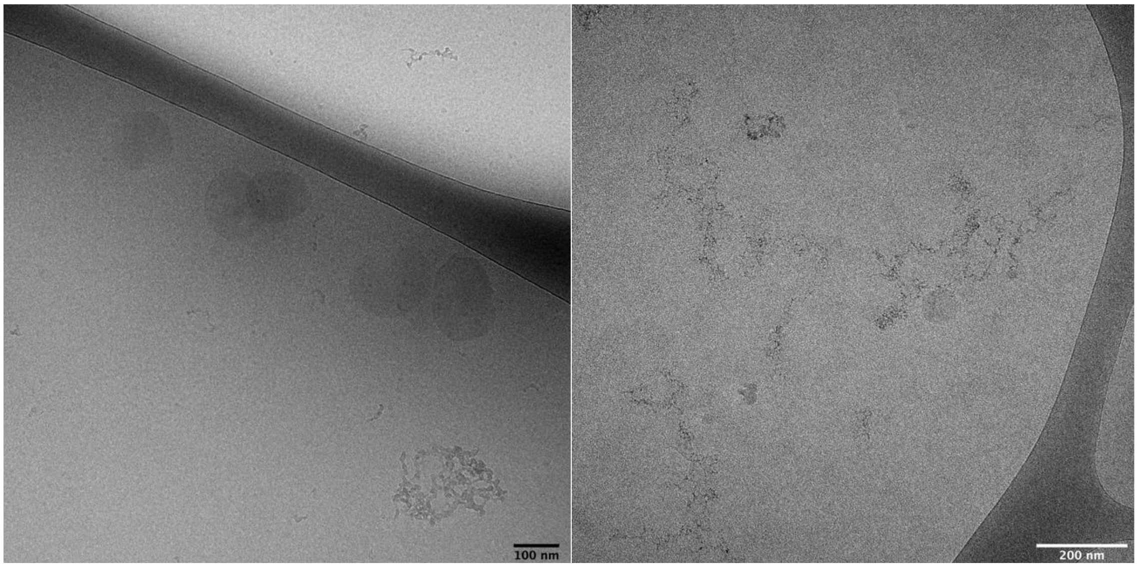


**Figure S5.** Cryo-TEM images of PNM2-16 incubated at 50 °C prior to blotting at 10-fold dilution 1 mg/mL (left) and at the initial concentration ~10 mg/ml (right).

## I. SEC chromatograms in water mobile phase

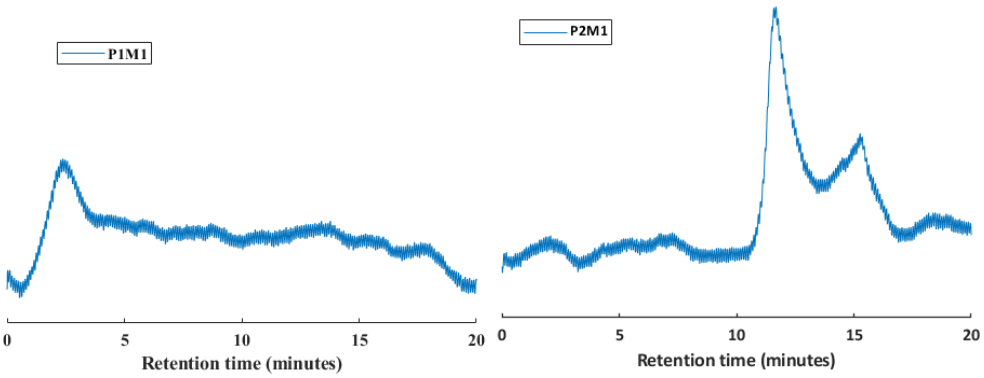


**Figure S6.** Inserts from the size exclusion chromatograms for sample PNM1-08 and PNM2-16.

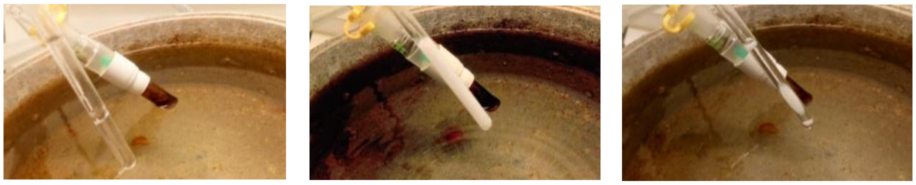

**Figure S7.** (Left) The PolyNIPAM (PN) solution at room temperature, (Center) the PN solution immediately after being submerged in a water bath at 35 ºC, (Right) the PN solution rapidly became clear again when removed from the water bath.

## J. Monitoring monomer consumption and composition by $^1$H NMR spectroscopy over time during the formation of sample PNM1-18 and PNM2-18

Time resolved spectra were analyzed in MestReNova V.9.0 (Mesterelab Research S.L.). Firstly, all the spectra were normalized to a reference peak using the artificial calibration signal generated from the ERETIC 2 (method applied for quantification, previously described in the experimental section). Then, the global spectral deconvolution (GSD) routine of the software was used for peak picking. From the results of the GSD, new spectra were obtained. These spectra were then used for line fitting on which all the calculations were based. Due to severe overlapping of signals from the NIPAM monomer and the glycomonomers, it was necessary to adjust the calculated integrals used to calculate compositions and conversions.

In the case of monomer conversion, the integral of the signals corresponding to the NIPAM monomer at 6.25-6.08 ppm (2 protons) and at 5.74-5.67 (1 proton) were used to calculate the consumption of the NIPAM monomer. However, these signals were overlapped by the signals

corresponding to the double bonds of the glycomonomer ($M_1EMA$ or $M_2EMA$) before polymerization. Therefore, the signal in the region 2.3-1.35 ppm, corresponding to the $–CH_3$ group of the glycomonomer at 1.92 ppm, was used to adjust the NIPAm integrals. Given the limitations of the $^1H$ NMR spectra due to the severe peak overlapping, we used this correction only up to the point of time when the signal at 1.92 ppm could no longer be fitted with the GSD and line fitting routines. Beyond this point this correction was not possible, and hence the integrals were used without further adjustment. It was not possible to determine with certainty when the glycomonomer was completely consumed due to peak overlapping. However, the peak at 1.92 ppm could not be fitted after 46 min in the polymerization for PNM1-18, and after only 19 min in the polymerization for PNM2-18. Even if there would be some unreacted glycomonomer left after these times, it was not possible to evaluate the residual amounts. However, it was clear that the glycomonomers polymerized at a considerably higher rate compared to NIPAm. Figure S8 displays the fitted data showing the conversion of NIPAm for PNM1-18 and PNM2-18.

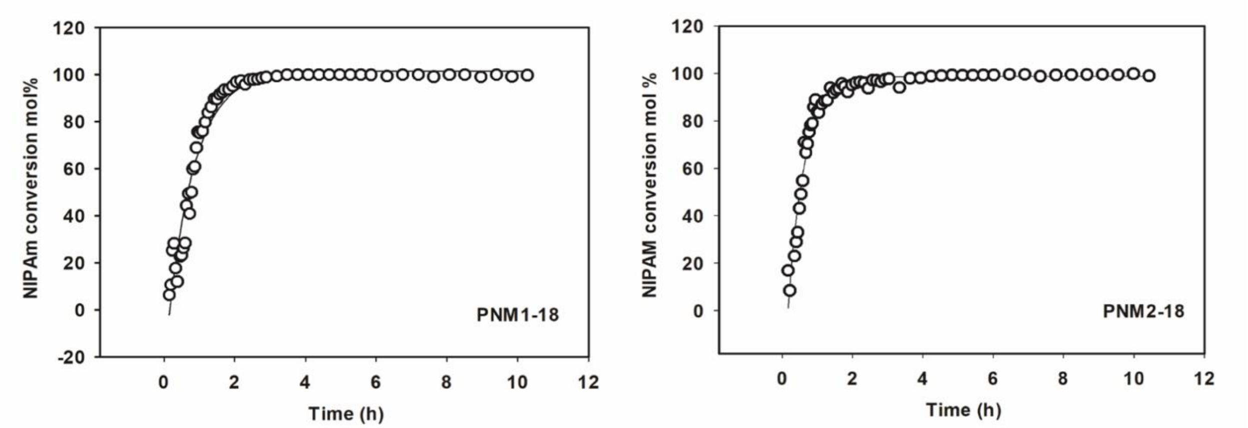


**Figure S8.** The conversion of NIPAm monomer over time calculated from $^1H$ NMR data taken over the course of the polymerization reactions to produce PNM1-18 and PNM2-18, respectively. Both polymerizations showed similar NIPAM conversion profiles.

Calculations of the comonomer composition was also attempted. In this case the signals employed to estimate the composition were, for the glycopolymer, the signal at 3.40-3.32 ppm assigned to 1 proton and, for NIPAm, the signal between 3.92 – 3.87 ppm that corresponds to 1 proton from polyNIPAm. The latter was adjusted by subtracting the integral equivalent of 1 proton from the glycopolymer that features an overlapping signal in the same region. Figure S9 shows the evolution of the composition over time for copolymer PNM1-18. Calculation of the corresponding data for PNM2-18 were impossible due to extensive peak overlapping, arising mainly from splitting resonances from signals of the glycopolymer in the regions suitable for composition calculation. Therefore, we did not report the composition evolution for this sample, but just reported the composition calculated for the final sample using the quantification method reported in the experimental section.

Figure S9 shows the evolution of composition of PNM1-18. At the beginning of the reaction the glycomonomer molar fraction is higher than that calculated in the final sample. Thus, confirming that there are differences in reactivity between NIPAm and the glycomonomer as previously

discussed. This would lead to the formation of gradient copolymers at the beginning of the polymerization as the glycomonomer is preferentially incorporated into the copolymer. Differences in the reactivity of monomers have been previously reported in copolymerizations of NIPAM with methyl methacrylate.[5]

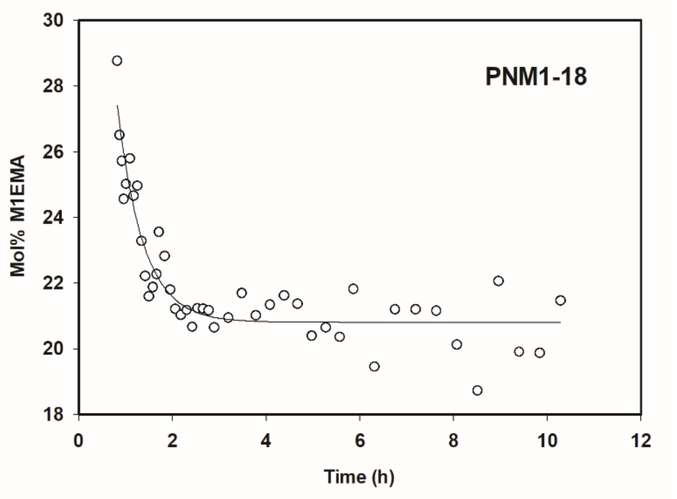


**Figure S9.** The evolution of composition over time calculated using $^1$H NMR data taken during the course of the polymerization reaction of PNM1-18.

## References


(1) Rosengren, A.; Butler, S. J. S. J.; Arcos-Hernandez, M.; Bergquist, K.-E. K.-E. E.; Jannasch, P.; Stålbrand, H. Enzymatic Synthesis and Polymerisation of β-Mannosyl Acrylates Produced from Renewable Hemicellulosic Glycans. *Green Chem.* **2019**, *21* (8), 2104–2118.

(2) Shen, Z.; Terao, K.; Maki, Y.; Dobashi, T.; Ma, G.; Yamamoto, T. Synthesis and Phase Behavior of Aqueous Poly(N-Isopropylacrylamide-Co-Acrylamide), Poly(N-Isopropylacrylamide-Co-N,N-Dimethylacrylamide) and Poly(N-Isopropylacrylamide-Co-2-Hydroxyethyl Methacrylate). *Colloid Polym. Sci.* **2006**, *284* (9), 1001–1007.

(3) Nyberg, N. RBNMR. MATLAB Central File Exchange 2020.

(4) Harris, R. K.; Becker, E. D.; De Menezes, S. M. C.; Granger, P.; Hoffman, R. E.; Zilm, K. W. Further Conventions for NMR Shielding and Chemical Shifts (IUPAC Recommendations 2008). *Magn. Reson. Chem.* **2008**, *46* (6), 582–598.

(5) Kim, J.; Choe, J.; Son, D.; Kim, M. Copolymerization Kinetics of a Simple Methacrylate and Functional Comonomers Via Cu(0)-mediated Reversible Deactivation Radical Polymerization. *Bull. Korean Chem. Soc.* **2019**, *40* (10), 1013–1019.
